# Review of Eukaryote Cellular Membrane Lipid Composition, with Special Attention to the Fatty Acids

**DOI:** 10.3390/ijms242115693

**Published:** 2023-10-28

**Authors:** Omeralfaroug Ali, András Szabó

**Affiliations:** 1 Agrobiotechnology and Precision Breeding for Food Security National Laboratory, Institute of Physiology and Animal Nutrition, Department of Animal Physiology and Health, Hungarian University of Agriculture and Life Sciences, Guba Sándor Str. 40, 7400 Kaposvár, Hungary; omeralfaroug.ali@gmail.com; 2 HUN-REN-MATE Mycotoxins in the Food Chain Research Group, Hungarian University of Agriculture and Life Sciences, Guba Sándor Str. 40, 7400 Kaposvár, Hungary

**Keywords:** membranes, phospholipids, sphingolipids, fatty acid, de novo synthesis, desaturation, oxygenation, bioactive lipids, physicochemical, very long polyunsaturated fatty acids

## Abstract

Biological membranes, primarily composed of lipids, envelop each living cell. The intricate composition and organization of membrane lipids, including the variety of fatty acids they encompass, serve a dynamic role in sustaining cellular structural integrity and functionality. Typically, modifications in lipid composition coincide with consequential alterations in universally significant signaling pathways. Exploring the various fatty acids, which serve as the foundational building blocks of membrane lipids, provides crucial insights into the underlying mechanisms governing a myriad of cellular processes, such as membrane fluidity, protein trafficking, signal transduction, intercellular communication, and the etiology of certain metabolic disorders. Furthermore, comprehending how alterations in the lipid composition, especially concerning the fatty acid profile, either contribute to or prevent the onset of pathological conditions stands as a compelling area of research. Hence, this review aims to meticulously introduce the intricacies of membrane lipids and their constituent fatty acids in a healthy organism, thereby illuminating their remarkable diversity and profound influence on cellular function. Furthermore, this review aspires to highlight some potential therapeutic targets for various pathological conditions that may be ameliorated through dietary fatty acid supplements. The initial section of this review expounds on the eukaryotic biomembranes and their complex lipids. Subsequent sections provide insights into the synthesis, membrane incorporation, and distribution of fatty acids across various fractions of membrane lipids. The last section highlights the functional significance of membrane-associated fatty acids and their innate capacity to shape the various cellular physiological responses.

## 1. Introduction

The biological membrane, commonly referred to as the biomembrane, holds paramount importance in both prokaryotic and eukaryotic cells. Its primary function lies in the selective regulation of molecular influx and efflux across the cellular boundary. Furthermore, it plays a crucial role in modulating intercellular communication and is involved in a vast array of complex processes, encompassing cell proliferation, differentiation, secretion, migration, invasion, and phagocytosis. However, the term “biomembrane” extends beyond the plasma membrane, as eukaryotic cells feature membranes within distinct cellular organelles [1,2], such as the endoplasmic reticulum (ER), mitochondria, nucleus, and various intracellular organelles. Additional functions of biomembranes revolve around stabilizing the consistency of cellular activities within the cell and organelles, controlling the trafficking of micromolecules (including O_2_, CO_2_, H_2_O, H^+^, K^+^, HCO_3_^−^, Mg^2+^, Ca^2+^, etc.) and macromolecular compounds, and providing a surface where essential biological events take place. According to Janmey and Kinnunen [3], biomembranes represent heterogeneous, asymmetrical bilayers with complex structures that contribute to the maintenance of cellular homeostasis and functionality. Hence, biomembranes’ systems exhibit considerable structural and dynamic diversity, making them an enduring area of scientific exploration.

The concept of Langmuir films, initially proposed by Langmuir in 1917, represents the earliest paradigm aimed at elucidating membrane systems [4]. Numerous subsequent paradigms have been developed in an attempt to explain membrane systems. The semifluid dynamics of biomembranes are merely determined by their intricate structure. The so-called “fluid mosaic model”, one of the most renowned models in the biological domain, is employed to illustrate the structure and function of membranes. Singer and Nicolson introduced this model in 1972 [5], describing lipids, proteins, and carbohydrates as the primary constituents of the membrane. In light of the fact that proteins do not actually dissolve in membrane lipids, this proposal has undergone several amendments. After 25 years of Singer and Nicolson’s proposal, Simons and Ikonen [6] proposed the “lipid raft” model, predicated on the clustering of sterols (namely, cholesterol in animals) and sphingolipids (SLs) within membranes to form microdomains where membrane-associated proteins are distributed. It has been established that these compartmentalized microdomains limit membrane lateral mobility and actively engage in various cellular events based on their structural arrangements [7]. Following the lipid rafts model, numerous other models have been introduced, which are either focused on revising the fluid mosaic model [8] or explaining the interaction between the similar [9] or distinct molecule classes [10] within membranes. Generally, the complexity of membranes exceeds that of model membranes due to the heterogeneous distribution of building molecules and their complex interactions. The continuous advancement of technology empowers science to delve deeper into the intricate structures of membranes, implying that the cell membrane model will invariably evolve toward increasing complexity, mirroring the progression from initial notions of membrane structure.

**Figure 1:**
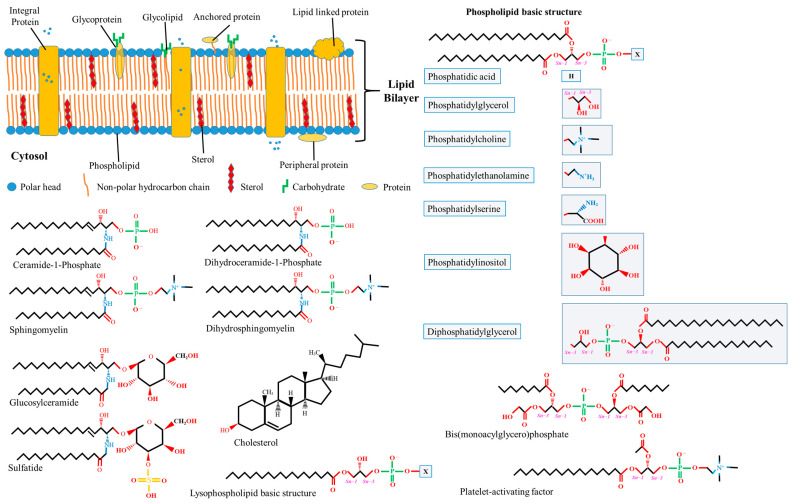
Schematic representation of biological compartments of the cell membrane and the molecular structure of the different lipids available in membranes. The molecular structures of different lipids have been adapted from the PubChem database (an open database for the public, available at https://pubchem.ncbi.nlm.nih.gov, accessed on 17 September 2023). Abbreviation: *Sn*, stereospecific numbering in the glycerol; 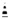 and 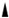, chiral carbon centers.

Lipids, proteins, and carbohydrates are pivotal biomolecules within biomembranes, exhibiting heterogeneous dispersion across membranes’ structures (see Figure 1). Membrane lipids, marked by diversity and possessing distinct properties either individually or in conjunction with other moieties, contribute to bilayer development and serve essential functions. Almost 50% of the membrane matrix is composed of proteins, which exist in various structures such as including integral (embedded with lipid bilayers), peripheral (associated with the membrane surface), and anchoring (not directly attached but rather bound to lipid embedded with lipid bilayers) proteins. Hydrophobic forces or ionic interactions mediate the binding of membrane proteins to membrane lipids, forming lateral domains with certain functions such as environmental communication, adhesion, trafficking, and signaling. Carbohydrates form covalent bonds with proteins or lipids within membranes, which only occur at the outward surface of the plasma membrane, yielding glycol-complexes [2,11]. The extant biotic assemblies within biomembranes are postulated to have transited from thermodynamic reactions on analogous abiotic assemblies [12]. The interaction between membrane lipids and proteins may modulate their individual qualities, thereby altering membrane conformation.

Among the constituents of biomembranes, fatty acids comprising the lipid portion have gained great focus due to their diverse functions in cellular processes. Understanding the diversity and composition of eukaryotic biomembrane lipids, especially fatty acids, is essential for elucidating the underlying mechanisms controlling cellular functions. Furthermore, it sheds light on the potential roles that particular lipids and fatty acids may play in various physiological and pathological processes, including inflammation and metabolic disorders. The current review primarily focuses on a healthy organism, intending to highlight the enormous diversity of biomembrane lipids and, as a secondary objective, characterize the biological roles of distinct fatty acids embedded into the cellular membranes. In addition, this review enhances our knowledge of fundamental cellular processes and subtly underscores the potential for therapeutic strategies centered on the lipid composition and fatty acid metabolism of biomembranes, which are likely promising foundations for further scientific inquiry.

## 2. Lipid Bilayer

Lipids have gained recognition and have become a subject of considerable interest among scientists since the original publication of Chevreul’s work [13], which delineated the concept of fatty acids. Lipids are widely acknowledged for their crucial role in forming cellular structures and mediating various physiological and life-sustaining processes. The concept popularity of a lipid layer’s existence on the cell’s surface can be traced back to Overton’s reports between 1885 and 1899, although a comprehensive elucidation of the membrane structure did not emerge until 1925 [14]. It was Gorter and Grendel who, employing a Langmuir monolayer, initially identified the presence of a lipid bilayer within blood chromocytes. Their discovery revealed a distinctive 2:1 ratio between the cellular surfaces covered by lipids and the estimated total cell surface area [15]. Consequently, a lipid bilayer emerges as a supramolecular matrix comprising two leaflets of lipid molecules residing within the biomembrane. Each leaflet necessitates a specific lipid composition characterized by certain physicochemical properties to finely modulate targeted functions.

Despite enduring exposure to changing conditions of temperature, pressure, and solvents, the lipid composition of animal cell membranes remains relatively stable, indicating a relatively confined capacity for drastic alterations in response to external stimuli. Nonetheless, the layers of membranes remain far from static; elements can transfer within (lateral diffusion) and between (vertical or flip-flop diffusion) leaflets. Lipid transporter proteins, namely, flippase, floppase, and scramblase, mediate the movement of lipids across membrane layers. In contrast, the retrograde traffic is responsible for the backward movement of lipids from membranes to organelles [2,16]. The ER, mitochondria, and Golgi apparatus are responsible for biosynthesizing most of the lipid classes in biomembranes, including glycerophospholipids, cholesterols (CHOL), and SLs. Conversely, lipid hydrolysis transpires within the lysosome, specifically the intralysosomal luminal vesicles, where numerous water-soluble hydrolases are active [17,18,19]. Lipids are transported to lysosomes through endocytic and autophagocytic pathways. The products generated from lipid hydrolysis are either utilized within the cell or expelled via exocytosis at the plasma membrane.

Thousands of lipid structures have been identified in mammals [20], with the coexistence of hundreds within a single cell remaining a probable [21]. The chemical properties of membrane lipids are characterized by distinctive features. These include the head-group or backbone structure, hydrocarbon chain length, degree of unsaturation, the presence of chirality, ionization, chelating power, and lipid concentration. Nevertheless, lipid classification is not arbitrary and can be predicated on physical properties, chemical properties, or biosynthetic qualities [22,23]. Within mammalian cell membranes, the preponderant lipid class is glycerophospholipids, also known as phospholipids. Characterized by a hydrophilic head group lining surfaces and a hydrophobic tail interposed in between, this class constitutes the bulk of the membrane lipid matrix. Other minor lipid classes recognized within biomembranes include glycolipids and sterols, with plasma membranes distinctively characterized by a considerable abundance of sterols. A schematic representation delineating the principal lipid classes identified in biomembranes is available in Figure 1. It is well-established that the lipid composition of biomembranes exhibits variations across organelles [24,25] and tissues; it dynamically adapts within the cell in response to specific cellular activities. The distinctive biophysical state of membrane lipids and the fatty acid composition may influence membrane rigidity, serve specific functions, and reveal the cell’s physiological state.

### 2.1. Glycerophospholipids

In 1811, the pioneering work of Vauquelin led to the identification of phosphorus in cerebral lipid extracts [26], and since then, phosphorus-containing lipids have become an intriguing field of investigation. This class of polar lipids is commonly referred to as ‘glycerophospholipids’ or simply “phospholipids”. It is the most prevalent lipid class in mammalian membranes, accounting for 50–60 mol% of the overall membrane lipid matrix [27]. The foundational structure of phospholipids closely resembles that of diacylglycerol (DAG, featuring a glycerol backbone with two acyl (fatty acid) chains at *sn*-1 and *sn*-2 positions); it is further distinguished by the inclusion of a polar phosphorus group at the *sn*-3 position. Hence, lipids within this class exhibit amphipathic properties, which are characterized by the presence of a hydrophilic head group and two hydrophobic fatty acids.

Over the past century, a multitude of phospholipid types have been identified, with variations in lipid structure playing a profound role in the differentiation of phospholipid varieties. The bulk of phosphate groups are attached to specific molecules or moieties, determining the exact type of phospholipid and its position within the lipid bilayer. Numerous phospholipids have been identified in mammalian membranes, including phosphatidic acid (PA), phosphatidylglycerol (PG), phosphatidylcholine (PC), phosphatidylethanolamine (PE), phosphatidylserine (PS), phosphatidylinositol (PI), diphosphatidylglycerol (DPG), bis(monoacylglycero)phosphate (BMP), platelet-activating factor (PAF), and lysophospholipids (LysoP).

#### 2.1.1. Phosphatidic Acid

**Figure 2:**
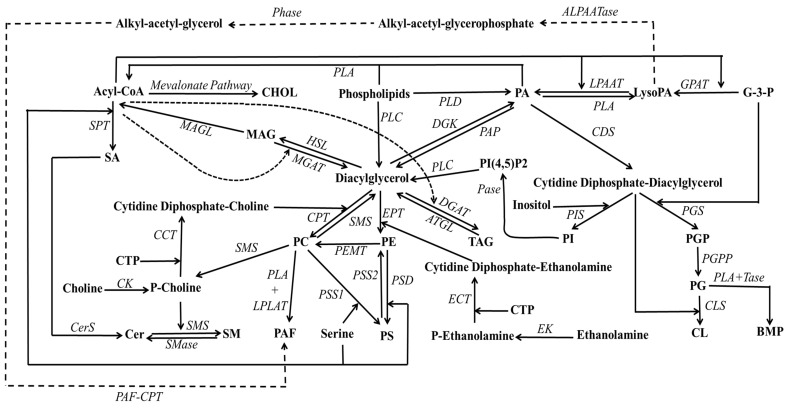
Schematic representation of the synthesis pathways for various phospholipids available in biomembranes. Abbreviations: ALPAATase, alkyl-acetyltransferase; ATGL, triacylglycerol lipase; CCT, cytidine 5′-triphosphate:phosphocholine cytidylyltransferase; CDP, cytidine diphosphate; CDS, CDP-DAG synthase; Cer, ceramide; CerS, ceramide synthase; CHOL, cholesterol; CK, choline kinase; CL, cardiolipin; CLS, cardiolipin synthase; CPT, CDP-choline:DAG cholinephosphotransferase; CTP, cytidine 5′-triphosphate; DG, diacylgelycerol; DGAT, DAG acyltransferase; DGK, DAG kinase; ECT, cytidine 5′-triphosphate:phosphoethanolamine cytidylyltransferase; EK, ethanolamine kinase; EPT, CDP-ethanolamine:DAG ethanolaminephosphotransferase; G-3-P, glycerol-3-phosphate; GPAT, glycerophosphate acyltransferase; HSL, hormone sensitive lipase; LysoPA, lysophosphatidic acid; LPAAT, lysophosphatidic acid acyltransferase; MAG, monoacylglycerol; MAGL, monoacylglycerol lipase, MGAT, monocylglycerol acyltransferase; P-Choline, phosphocholine; P-ethanolamine, phosphoethanolamine; PA, phosphatidic acid; PAF, platelet activating factor; PAF-CPT, platelet activating factor cholinephosphotransferase; PAP, phosphatidic acid phosphatase; Pases, phosphatases; PC, phosphatidylcholine; PE, phosphatidylethanolamine; PEMT, phosphatidylethanolamine *N*-methyltransferase; PG, phosphatidylglycerol; PGP, phosphatidylglycerophosphate; PGPP, phosphatidylglycerophosphate phosphatase; PGS, phosphatidylglycerophosphate synthase; Phase, phosphohydrolase; PI, phosphatidylinositol; PIS, phosphatidylinositol synthase; PLA, phospholipase; PLC, phospholipase C; PLD, phospholipase D; PS, phosphatidylserine; PSD, phosphatidylserine decarboxylase; PSS, phosphatidylserine synthase; SA, sphinganine; SM, sphingomyelin; SMase, sphingomyelinase; SMS, sphingomyelin synthase; SPT, serine palmitoyltransferase; TAG, triacylglycerol; Tase, transacylase.

PA, often referred to as phosphatidate (see Figure 1), represents the simplest phospholipid structure and tends to accumulate in membranes in relatively minor proportions, owing to the activity of lipid phosphate phosphohydrolases [28,29]. It was initially identified as a phosphorylated isomer of DAG [30]. PA, therefore, constitutes a non-bilayer lipid characterized by a phosphate group esterified at the *sn*-3-hydroxyl of the glycerol backbone and two fatty acyl chains occupying the remaining *sn*-positions. Multiple pathways contribute to PA production (see Figure 2), including the dual acylations of glycerol-3-phosphate, phospholipid hydrolysis pathway (especially involving PC), and DAG phosphorylation [31]. The synthesis of PA from DAG is a reversible process catalyzed by DAG kinase and PA phosphatases (also referred to as lipins).

PA is a negatively charged anionic lipid involved in cellular signal transduction and capable of reacting with divalent ions such as Ca^2+^. Furthermore, its presence within mammalian cells is vital, as it acts as a mediator for phospholipid metabolism, a regulator for glycerolipid metabolism, neuroendocrine cell exocytosis, protein kinases, small G-proteins, and a modulator for membrane fusion and fission machinery [32,33,34,35,36]. Therefore, any alterations in PA levels may indicate disruptions in cellular homeostasis and the onset of metabolic and health-related consequences, as evidenced by Tanguy et al. [31], who linked the high accumulation of PA in cells to metabolic disorders.

#### 2.1.2. Phosphatidylglycerol

When alcohol glycerol esterifies with a phosphate within a phospholipid, the resulting lipid structure is referred to as ‘PG’. Benson and Maruo identified this lipid structure in 1958 [37]; it is characterized by two free hydroxyl groups. Basically, it comprises a glycerol backbone linked with two fatty acyl chains and phosphoglycerol. Within mammals, PG is synthesized in the mitochondria through multiple pathways: (1) it originates from imported PA, which undergoes a series of enzymatic reactions involving intermediates within the cytidine diphosphate-diacyl glycerol pathways in the inner mitochondrial membrane, and (2) from dephosphorylated phosphatidylglycerolphosphate catalyzed by the mitochondrial phosphatase enzyme [38].

Though PG does not constitute a substantial proportion of mammalian membranes (1–2% of membrane polar lipids), it accounts for up to 7–15% of the lipid composition in lung surfactants [39,40]. This heightened presence of PG in the lungs, where it ranks as the second most prevalent phospholipid in the lungs, underscores its crucial role in surfactant activity. Beyond the lung, the PG functionality extends to lipid–protein and lipid–lipid interactions, along with its influence on membrane rigidity. The PG molecular structure relatively resembles that of DPG and BMP, with all of them featuring more than glycerol in their structures. Furthermore, the molecular structure of PG in specific tissues has been considered to be a functional analogue of PI (having an inositol group rather than glycerol) [41]. Thus, these phospholipids may manifest similar activities, such as the inhibition of phosphatidylcholine-dependent kinase activity in swine brain [42]. Elevated levels of PG have been associated with viral infection, as PG can integrate into viral membranes during replication [43,44,45]. In contrast, some reports suggest that PG is involved in regulating innate immunity and suppressing viral infection [46,47,48], potentially including COVID-19 infection [49]. Therefore, further studies are imperative to ascertain the significant biological roles of PG in various mammalian species.

#### 2.1.3. Phosphatidylcholine

The PC, also known as lecithin, was the first isolated phospholipid in 1850, with choline (a source of the methyl group) serving as the polar head [50]. Herein, it is very self-evident that the PC structure is not entirely endogenous, as choline is an essential nutrient for mammals. PC is a ubiquitous presence in all cell membranes, spanning prokaryotic cells (e.g., bacteria) and eukaryotic cells (i.e., cells of plants and animals). Structurally, PC exhibits two major linkage types in tissues: diacyl-PC (ester bond; most abundant in eukaryotes) and alkyl-PC (featuring an ether bond) [51]. Additionally, the less common isomer of PC is alkenyl-PC (vinyl ether bond), which is referred to as choline plasmalogens and plasmenylcholine. These lipids typically comprise two fatty acids linked to glycerol through ether and ester bonds at *sn*-1 and *sn*-2, respectively [52,53].

PC represents the most abundant phospholipid class (constituting nearly 50% of all phospholipids within bilayers), particularly in the pulmonary surfactant, where dipalmitoyl-PC predominates [54,55,56,57]. As a fundamental building block of the membrane bilayer, PC occupies the outer leaflet [58]. Remarkably, approximately 80 to 90% of the lipids in the plasma membrane’s outer leaflet consist of PCs. The preponderance of PC synthesis occurs in the ER, where cytidine 5′-triphosphate (CTP):phosphocholine cytidylyltransfease (PCT) (generally known as CCT) [59,60] catalyzes the rate-limiting step in the cytidine 5′-diphosphocholine (CDP-choline, citicoline or Kennedy) pathway [61]. This CDP moiety is not only involved in PC biosynthesis [62] but in all other phospholipids, with the exception of PA, depending on which moiety replaces choline. A distinctive pathway for PC biosynthesis exclusively takes place in the liver, where PC is generated from PE via sequential methylation [63], facilitated by the phosphatidylethanolamine *N*-methyltransferase (PEMT) that is found in the mitochondrial-associated membranes (MAM).

It has been believed that PC’s relatively neutral molecular properties (having positive and negative charges but lacking net charge) and its predominance play an essential role in maintaining biomembrane integrity and functionality. Unlike other phospholipids, PC does not exhibit negative charge repulsion. PC serves as a precursor for sphingomyelin (SM) due to its choline molecule [64]. In addition, it acts as a precursor for other polar lipids, such as PA, lysophosphatidylcholines (LysoPC), PS, and PAF. PC plays a crucial part in cell signaling processes and impacts the concentration of circulating lipoproteins [56,65,66]. Furthermore, it is integral to membrane trafficking and molecule transportation. LysoPC composed of C22:6 (at the *sn*-2 position) has been demonstrated to be more effective than C22:6-free fatty acids in crossing the blood–brain barrier [67].

#### 2.1.4. Phosphatidylethanolamine

**Figure 3:**
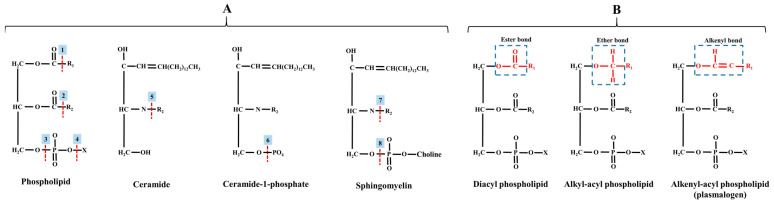
(**A**) Site activities of different phospholipases on membrane lipids. (**B**) Different linkage types in phospholipids. Abbreviations: 1, phospholipase A1; 2, phospholipase A2; 3, phospholipase C; 4, phospholipase D; 5, ceramidase; 6, lipid phosphate phosphatase; 7, sphingomyelin deacylase; 8, sphingomyelinase; R, fatty acid; X, head group.

Following PC, the second most prevalent phospholipid in mammalian tissues is PE, formerly known as “cephalin”. It was the second discovered phospholipid in cerebral tissue by Thudichum in 1884 [68], constituting approximately 15–25% of the total phospholipids in mammalian cell membranes [69]. In neural tissues, PE can reach even higher levels, up to 45% [70], pointing out its essential role in this tissue. It is profoundly abundant in mitochondrial membranes and is exclusively localized in the cytosolic leaflet of the plasma membrane, in contrast to PC [58]. The structure of PE involves the esterification of the phosphatidyl group to the hydroxyl group of an amino group (namely, the ethanolamine), resulting in a small reactive head group. PE does not form a bilayer independently (on its own) but exhibits an inverted hexagonal phase. This class of lipids features various linkages, including diacyl, alkyl, and alkenyl configurations (see Figure 3). Ethanolamine plasmaologens, also known as plasmenylethanolamine, are more abundant than plasmenylcholine in many tissue types, except for the heart and smooth muscle [52].

In eukaryotes, the biosynthesis of PE is an outcome of multiple pathways, notably, the de novo synthesis of PE through CDP-ethanolamine [61] and the salvage pathway involving the decarboxylation of PS by phosphatidylserine decarboxylase (PSD) in the mitochondria [71]. Additional pathways involved in the remodeling of PE, which are also identified in bacteria and plants, include the following: (1) the base-exchange pathway between PE and PS [72]; (2) the degradation of sphingosine-P via sphingosine-P lyase [73]; and (3) the reacylation of Lyso-PE at MAM [74]. Notably, despite the structural resemblance of PE and PC, PE exhibits distinct chemical and biological properties. PE stands apart from PC due to its smaller head group, which manifests less affinity to water. Consequently, PE can form compact aggregation and displays a heightened thermostability [75,76]. These attributes significantly contribute to membrane rigidity, making PE an indispensable component of the membrane’s architecture, permeability, and fluidity.

In terms of membrane rigidity, PE often mimics the behavior of CHOL, particularly in insects [77]. In light of these findings, the PC/PE ratio is likely to exert a substantial influence on the functionality, fluidity, stability [78], and permeability of the membrane. Furthermore, PE plays a vital role in upholding membrane integrity and participating in cellular signaling. Studies have revealed that PE is implicated in various processes, including membrane-to-membrane fusion [79], DAG generation through the involvement of phospholipase C (PLC), and the modification of membrane proteins through the mediation of reactive aldehydes [80]. PE has also been observed to induce negative curvature in biomembranes [81], which is primarily attributed to its diminutive conical head group. In addition, PE serves as a precursor for various other lipids, including *N*-acyl-phosphatidylethanolamine (NAPE), which serves as a crucial precursor during the biosynthesis of certain essential biological compounds in the brain (e.g., anandamide) [82,83].

#### 2.1.5. Phosphatidylserine

Folch and Schneider identified serine in cephalin components in 1941 [84], marking the beginning of the discovery of PS. PS is a minor class of phospholipids in mammalian cells (2–15% of total phospholipids), which demonstrates a pronounced tendency for accumulation within the cerebral cortex [85,86]. It has also been detected in the membranes of organelles such as mitochondria and ER, where it serves as a substrate for the production of PE. Notably, the distinguishing feature of the PS structure, setting it apart from other phospholipids, is the attachment of the serine residue to the phosphatidyl group, resulting in the formation of a negatively charged head group. This characteristic renders it exceptionally reactive with divalent metals. In contrast to PC and PE, PS exclusively exists in a diacyl isomer, with *sn*-2 being markedly unsaturated [85].

In contrast to plants [87], yeasts, and prokaryotes [88], mammalian cells lack the de novo CDP-DAG biosynthetic pathway for PS biosynthesis. The biosynthesis of PS in mammalian cells transpires both in the MAM and in the cytosol of the ER and is facilitated by a calcium-dependent base exchange. This pathway is catalyzed by PS synthase-1 and -2 (PSS1 and PSS2, respectively), utilizing PC (catalyzed by PSS2) and PE (catalyzed by PSS2) as the primary precursors at both sites [89]. Subsequent to its production, a fraction of PS translocates to the plasma membrane via passive diffusion. This lipid primarily localizes to the cytosolic leaflet of the plasma membrane [90], although its migration to the outer leaflet is notable during programmed cell death [91] and cancer progression [92].

The externalization of PS on the cell’s outer layer serves as a molecular signal, prompting neighboring cells, including macrophages, to engulf and phagocytose the dying cell. Beyond this role, PS plays a multifaceted biological role within cellular functions. It contributes to the recognition and communication mechanisms between cells. PS existence is crucial during PE biosynthesis, acting as a source pool [71]. Furthermore, PS has been observed to interact with SLs, resulting in elevated interdigitation under the influence of CHOL [93]. PS is also vital for the maintenance of plasma membrane integrity within mammalian cells, exerting modulation over membrane fluidity and permeability, both of which are essential for the regular function of membrane-bound proteins.

PS has been implicated in the activation of protein kinase, prothrombinase, and neuroinflammation signaling pathways, as well as being an essential element of lipid–calcium–phosphate complexes [94,95,96,97]. Consequently, PS facilitates a range of membrane-bound signaling processes, including apoptosis, activation of enzymes, immune regulation, coagulation cascade, and mineral deposition during bone regeneration.

#### 2.1.6. Phosphatidylinositol

The earliest documented report of phosphatidylinositol (PI) traces back to the year 1930 when inositol was initially identified within a lipid extract [98]. It was not until nearly three decades later, in 1959, that Pizer and Ballou elucidated the precise structure of PI [99]. PI, an anionic phospholipid, features a distinctive inositol head group, characterized by a hexa-hydroxy-ring consisting of six carbon atoms. Within the realm of inositol-containing phospholipids (phosphoinositides), PI represents the most elementary form, with the other seven isomers constituting phosphorylated derivatives of the PI structure [100]. In eukaryotic organisms, three primary forms of phosphoinositides prevail: (1) PI, formerly recognized as monophosphoinositide, in which 1′-myo-inositol is linked to PA; (2) PI4P, where a phosphate group esterifies position 4 of inositol, formerly referred to as diphosphoinositide; and (3) PI5P, featuring a phosphate esterifies position 5 of inositol. In eukaryotes, the phosphorylation of positions 2 and 6 of PI is impeded due to steric hindrance. PI can constitute up to 10% of total phospholipids and is ubiquitously present in the cytosol of all cellular membranes and certain organelles (e.g., endoplasmic reticulum and Golgi apparatus) [101,102]. PI of eukaryotic organisms is primarily biosynthesized from PA via a de novo pathway and is catalyzed by the CDP-DAG synthase (which serves as a rate-limiting enzyme [103]) and CDP-DAG myo-inositol 3-phosphatidyltransferase [104]. These enzymes are localized in the ER, where they facilitate the formation of intermediates from PA and the attachment of myo-inositol to these intermediates, respectively. Mammalian cells possess the capability to synthesize inositol de novo from glucose-6-phosphate. Other marked three biosynthetic pathways have been identified in plants and prokaryotes, with the most recent discovery occurring a decade ago [105].

Though PI’s initial discovery was nearly a century ago, our understanding of the biological functions of PI has markedly advanced over the past three decades. PI is not merely a component of bilayer lipids; it is involved in various metabolic processes [106]. Its significance extends to the brain, where it serves critical functions. In addition, it serves as the primary pool of the C20:4 n6 fatty acyl chain in animal cells, frequently occupying the *sn*-2 position [107,108,109]. This specific acyl chain is of paramount importance for the biosynthesis of eicosanoids, including prostaglandins [101,110]. The enzyme phospholipase A2 (PLA2) is responsible for the removal of C20:4 n6 from PI, resulting in the formation of LysoPI (see Figure 3). Consequently, an accumulation of LysoPI indicates heightened PLA2 activity, implying metabolic alterations and, potentially, the progression of cancer [111].

Furthermore, PI constitutes the major substrate of the signaling DAG in mammalian cells, a process catalyzed by the PLA2 and PLC enzymes. Thus, PI emerges as a dynamic lipid that participates in intracellular signaling, inflammation, and immune regulation. PI also contributes to the formation of glycosyl bridges that facilitate the binding of multiple proteins (known as glycosyl-phosphatidylinositol (GPI)-anchored proteins) to the cellular membrane surface [112]. PI has been shown to engage in regulating protein activities at the cellular interface. The various phosphoinositides, including PI3P, PI4P, PI5P, PI(4,5)P2 (the most abundant PI-phosphorylated structure in mammalian membranes), PI(3,4)P2, PI(3,5)P2, and PI(3,4,5)P3, while accumulating in very low concentrations (1%), significantly contribute to membrane organization. An in-depth discussion has been reviewed by Posor et al. [113]. For instance, PI(4,5)P2 functions as a cofactor for phospholipase D (PLD), an enzyme responsible for the production of PA, which serves as a signaling molecule.

#### 2.1.7. Diphosphatidylglycerol

The DPG, also known as cardiolipin (CL), was initially isolated from bovine hearts by Pangborn in 1942 [114], and the nomenclature “cardio” reflects its association with cardiac tissues. This uncommon tetra-acylated phospholipid is exclusively confined to the inner and outer mitochondrial membranes, with the production site on the matrix side of the mitochondrial inner leaflet [115]. Basically, it can be described as PG with additional phosphatidate groups esterified to glycerol, resulting in two negative charges. The biosynthesis of CL primarily proceeds from the PA substrate [116], which is subsequently transformed into PG within the mitochondria. The conversion of PG species into CL through condensation is facilitated by cardiolipin synthase (CLs). It is postulated that the biosynthesis of CL in eukaryotic cells has evolved from prokaryotic ancestors [117].

CL plays a pivotal role in mitochondrial activity, which is evident through its substantial concentration (15–20%) among the total polar lipids of the mitochondria [118]. Thus, it dynamically contributes to the respiratory chain, interacts with adenosine diphosphate (ADP)/adenosine triphosphate (ATP) and imported complex III and IV proteins, regulates mitochondrial fission and fusion, and controls the release of apoptotic factors [119,120,121]. Therefore, variations in CL concentrations may be associated with mitochondrial dysfunction disorders [119].

#### 2.1.8. Bis(monoacylglycero)phosphate

The BMP is a unique lipid involved in cellular trafficking due to its enrichment in the intracellular membranes of the late endosomes [122,123] and lysosomes [124]. Body and Gary were the first to isolate it from pig lungs in 1967 [125]. While it was initially misidentified as “bisphosphatidic acid” or “lysobisphosphatidic acid”, BMP’s accurate structural characterization was reported by Brotherus and Renkonen in in vitro cultured hamster kidney fibroblast cells [126]. BMP is a negatively charged structural isomer of PG, featuring an unusual *sn*-1-glycerophospho-*sn*-1′-glycerol configuration. This lipid structure is related to polyglycerophospholipids, which also encompass PG and DPG [85]. In fact, PG has been identified as the substrate for BMP production [127,128,129], though the precise mechanisms of their production and metabolism continue to be subjects of ongoing research. PG is a fundamental component of mitochondria and ER, and it reaches the lysosome (the BMP biosynthesis site) via autophagy. Herein, the phospholipases are less effective towards BMP, preventing the lysosomal membranes from autodigestion.

The production of BMP involves multiple reactions, including the acylation of fatty acid to glycerol’s hydroxyl moiety and the esterification of phosphoric acid to ethanolamine. Despite BMP constituting a minor fraction of cellular polar lipids, comprising less than 1% of the total [130], elevated levels have been detected in rat splenic tissue [131] and alveolar macrophages [132]. Elevated BMP concentrations have been associated with lipid storage diseases and drug-induced lipidosis [132,133,134]. Studies on BMP have consistently increased over the past 14 years. This interest is attributed to its role in the metabolism of glycosphingolipids (GSLs) and CHOL [19,135], which, in return, influence cellular signaling, vesicle fusion, and membrane integrity.

#### 2.1.9. Platelet-Activating Factor

The PAF is a unique bioactive ether phospholipid with a structural composition of 1-alkyl-2-acetyl-*sn*-glycero-3-phosphocholine structure, notably lacking the conventional phospholipid *sn*-1 ester bond [136]. It was initially introduced by Benveniste et al. [137] from rabbit platelets, making PAF the earliest identified phospholipid capable of inciting an inflammatory response. The biosynthesis of PAF occurs within the ER through two primary pathways: the de novo pathway from PC transferred to alkyl acetyl glycerol [138] and a biomembrane remodeling process that involves the substitution of the *sn*-2 fatty acyl chain with an acetyl group [139]. The latter pathway is catalyzed by PLA2 and acetyltransferase/transacylase.

The accumulation of PAF exhibits variations among cell types, typically representing a negligible fraction of the total phospholipids. This characteristic poses challenges in its precise quantification. The heightened accumulation rate of PAF observed in various tissues correlates with inflammatory responses [140], projecting its major involvement in the regulation of inflammatory and immune responses, as well as physiological processes such as platelet aggregation and thrombosis. In addition, PAF has been documented to exert influence over the activities of multiple physiological systems, including the cardiovascular, nervous, respiratory, excretory, and reproductive apparatuses [141,142,143]. However, alterations in PAF concentrations have been associated with certain diseases, syndromes, and skin cancer [144,145], albeit without serving as a direct mediator.

#### 2.1.10. Lyso-lipids

Shifting the focus to lyso-lipids, this class is alternatively referred to as hydrolyzed lipids. This lipid class is constituted by various isomers originating from the enzymatic cleavage of acyl chains from phospholipids or SLs, which are catalyzed by phospholipase and deacylase enzymes, as illustrated in Figure 3. Thus, lyso-lipids of membranes can be categorized according to their original backbone and further classified into lysoglycerophospholipids (LysoPs) and lysosphingolipids (LsoSLs), respectively. Generally, LysoPs are amphipathic molecules carrying either an alkyl or acyl chain [146,147]. On the other hand, the LysoSLs are distinct due to the absence of the amide-linked fatty acid at the 2-amino position of the sphingoid base [148], setting them apart from their parental structure. Long ago, LysoP isomers were considered intermediates in phospholipid biosynthesis or fragments of disrupted cells. Nevertheless, they display distinct properties and functions compared to parental phospholipids. LysoP contributes to cellular homeostasis by engaging in bilayer remodeling and rigidity. Furthermore, specific LysoP molecules can function as ligands for various G-protein-coupled receptors [149], underscoring their involvement in cellular signaling.

While the current review does not emphasize this category due to its limited prevalence and identification in studies, the most abundant LysoP class is lysophosphatidylcholines/lysolecithins (LysoPC). LysoPC is generated through the hydrolysis PC, mainly catalyzed by PLA2. LysoPC possesses physical properties distinct from PC, forming micelles rather than bilayers. It is typically found in minute proportions and plays a role in the mechanism of the autoimmune response [150]. The accumulation of LysoPC within cells has been associated with metabolic irregularities [150] and phospholipid peroxidation [151,152]. Lysophosphatidic acid (LysoPA), the simplest structure within the LysoP category within mammalian membranes, is biosynthesized in most cells through the activity of lysophospholipase-D on LysoPC or via the actions of phospholipases (phospholipase A1 (PLA1) and PLA2) on PA [153]. LysoPA serves numerous functions, including the regulation of cellular differentiation, growth, proliferation, migration, and apoptosis. In the context of inflammation and cancer, it has gained significant attention, focusing on its structural features and the extent of accumulation [154,155].

### 2.2. Sterols

This category of membrane lipids is named according to its primary constituent, sterol. Alternatively, it can be referred to as steroid alcohol, distinguishing it from phospholipids. Sterols are characterized by a rigid, always *trans* tetracyclic hydrocarbon ring, a 3β-hydroxyl group, and a flexible side fatty acyl chain as a tail [156]. Thus, sterols possess both hydrophilic properties (represented by the hydroxyl group) and hydrophobic properties (attributed to the ring and fatty acyl chain). Notably, variations in the floppy tail of sterols account for the structural diversity observed across different biological kingdoms. Sterols are primarily found in membranes of animals (cholesterol), plants (stigmasterol or β-sitosterol), and fungi (ergosterol). It is important to note that most bacterial membranes are devoid of sterols. Among mammalian membranes, CHOL is the most commonly encountered sterol and recognized structure. Despite its widespread presence in various organisms, it is noteworthy that certain insect species lack the genes responsible for its biosynthesis [157].

#### Cholesterol

CHOL is a sterol isoprenoid characterized by a semi-rigid tetracyclic ring composed of three six-membered rings and one five-membered ring. It features a polar 3β-hydroxyl group and an 8-carbon chain attached to the carbon-17 position, while its side acyl chain exhibits conformational flexibility [158,159]. The polar nature of the CHOL group alone prevents CHOL from forming bilayers. However, when synthetic CHOL is combined with a PC head moiety, bilayer formation occurs [160]. Within bilayers, CHOL is asymmetrically distributed, with the majority of sterols (60–70%) located in the inner leaflet [161,162]. CHOL was initially discovered in gallstones by de La Salle in 1858, but it took another decade for researchers to identify it [163].

CHOL can be obtained from the diet or synthesized by the liver (which contributes 50% to total CHOL synthesis) and the ERs of other cells. The biosynthesis of CHOL is regulated by sterol-responsive element binding protein 2 (SREBP2)-cleavage-activating protein, which senses intracellular CHOL and modulates nuclear transcription. Importantly, cells can also import CHOL from the vascular system, where lysosomes recycle the low-density lipoprotein by transferring CHOL to the ER. The CHOL biosynthesis pathway involves a series of enzyme-catalyzed reactions generating a series of intermediate compounds. Typically, over 20 enzymes are involved in the CHOL biosynthesis pathway, using acetyl-CoA as a substrate. Though animal cholesterol is synthesized in the ER, a relatively higher proportion is found in cellular plasma membranes than in the ER [2,164]. Notably, the plasma membrane contains a significant amount of CHOL (making up to 50% of membrane lipids, primarily in the cytosolic leaflet) as compared to other subcellular organelles [162,165]. In the cytosolic leaflet, the hydroxyl group and the aliphatic chain are oriented towards the aqueous phase and the bilayer’s interior, respectively.

CHOL plays an important role in modulating dynamic membrane activities [156]. Its fused ring structure (exhibiting amphiphilic properties) imparts greater membrane rigidity. Thus, CHOL levels critically influence membranes’ rigidity, fluidity, and permeability [166,167]. The incorporation of CHOL into membranes reduces permeability to non-polar molecules while increasing the hydrophobic barriers to polar molecules. CHOL also has a condensing effect on hydrocarbon chains, thereby reducing the surface area occupied by lipids [168]. Additionally, CHOL participates in the formation of lipid rafts through interactions with various phospholipids, with a notably favorable interaction observed with saturated phospholipids [169]. The solubility of CHOL in membranes depends on the degree of unsaturation of the phospholipids. A high number of unsaturated double bonds has been shown to reduce CHOL solubility [170,171]. Remarkably, even among saturated phospholipids, CHOL affinity was shown to be different. CHOL’s affinity to other lipid complexes relies on various factors, such as the head group tilt structure [172], hydration [173], acyl chain order [174], possible interdigitation of acyl chains [175], and the presence of hydrogen bond acceptor and donor groups [176].

CHOL serves a wide range of signaling functions through its interactions with various cellular molecules and receptors. A recent study indicated that the interaction between cholesterol and lipid-mediated innate immune memory triggers cytokine cascades as associated with COVID-19 [177]. Conversely, an imbalance in membrane-CHOL levels may likely pose severe metabolic consequences, including cancer progression [178,179]. CHOL also serves as a precursor for the biosynthesis of bile acids and steroid hormones [180,181], which mediate crucial roles in biological processes, such as carbohydrate metabolism [182,183,184]. Furthermore, CHOL esters play a critical role in transporting fatty acyl chains via lipoproteins in the bloodstream, and these esters are integral components of amphiphilic plasma lipoproteins [185].

### 2.3. Sphingolipids

SLs constitute a class of lipids that are present in cells in relatively lower proportions, typically accounting for about 10–20% of total cellular lipids. Despite their relatively lower abundance, SLs exhibit significant signaling activities [27]. The bio-functional roles of SLs can be broadly categorized into three areas: firstly, they modulate the physical properties of biomembranes; secondly, they serve as signaling molecules, acting as second messengers or secreted ligands for cell-surface receptors; and thirdly, they mediate interactions between cells and their external environment [186]. Hence, variations in the ratio of SLs can have a substantial impact on cellular activities and overall cellular survival. The initial identification of SLs can be attributed to Thudichum [68], while the elucidation of their structure, namely, the sphingosine (SO) component, was achieved by Carter [187]. Unlike phospholipids, which are glycerol-based, SLs consist of a long-chain sphingoid base as a backbone, with an amide-linked acyl chain attached instead of an oxygen ester. Notably, the hydrophobic chains (fatty acid) in sphingosine (SO) are structurally constant and non-hydrolyzable, distinguishing SLs from the variable and hydrolyzable fatty acids found in phospholipids. Numerous distinct SL structures have been identified, with structural differences primarily based on variations in backbone structure, hydrophobic chain length, and the level of unsaturation.

#### 2.3.1. Sphingoid Bases

**Figure 4:**
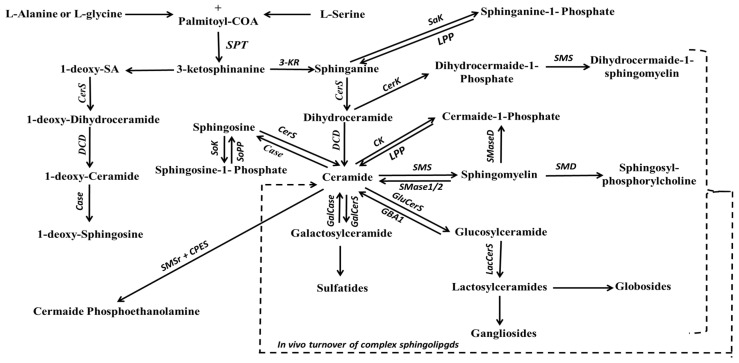
Schematic representation of the de novo biosynthesis pathway of major complex sphingolipids. Abbreviations: 3-KR, 3-ketosphinanine reductase; Case, ceramidase; CerK, ceramide kinase; CerS, ceramide synthase; CPES, ceramide phosphoethanolamine synthase; DCD, dihydroceramide desaturase; GalCase, galactosylceramidase; GalCerS, galactosylceramide synthase; GBA1, acid β-glucosidase; GluCerS, Glucosylceramide synthase; LacCerS, lactosylcermaide synthase; LPP, lipid phosphate phosphatase; Sak, sphinganine kinase; SMaseD; sphingomyelinaseD; SMD, sphingomyelin deacylase; SMS, sphingomyelin synthase; SMS1/2, sphingomyelin-1 or -2; SMSr, sphingomyelin synthase related proteins; Sok, sphingosine kinase; SoPP, sphingosine phosphate phosphatase; SPT, serine palmitoyltransferase.

Among the most well-known backbone structures are sphinganine (SA) and SO bases, which serve as the primary reservoir for SL biosynthesis. In the cytosolic side of the ER, serine palmitoyltransferase (SPT) catalyzes the condensation of palmitoyl-Coenzyme A with L-serine, resulting in the formation of 3-ketosphinganine [188]. Subsequently, under the influence of 3-ketosphinganine reductase, 3-ketosphinganine is transformed into SA (as shown in Figure 4). SPT, a pyridoxal 5′-phosphate-dependent enzyme, is the rate-limiting enzyme for SA production [189]. It is worth noting that SPT is not limited to serine alone as a substrate; studies have shown that it can also employ alanine and glycine [190], leading to the production of structures known as 1-deoxysphingolipids. On the other hand, SO is biosynthesized during ceramide (Cer) production or hydrolysis, a process catalyzed by delta-4-desaturase (∆4-desaturase, or D4D) and SPT enzymes, and ceramidase (CDase), respectively. However, it is important to highlight that free SO is not produced via the de novo pathway; rather, it is generated from the hydrolysis of Cer by CDase.

SA and SO kinases can phosphorylate SA and SO, generating their respective 1-phosphate forms/derivatives. This pathway is reversible, and sphingoid-1-phosphate produced in this manner can undergo dephosphorylation through sphingoid-1-phosphate phosphatases. Sphingoid-1-phosphate remarkably differs in its activities from free sphingoid bases, serving not only as second messengers but also as first messengers [191]. Sphingoid-1-phosphate also serves as a substrate for phospholipid synthesis, as well as having a universal cellular survival signal [192] that is mediated by its binding to specific G protein-coupled cell surface receptors [193]. Sphingosine phosphate lyase has the capacity to cleave sphingoid-1-phosphate into phosphatidylethanolamine [194,195].

On the other hand, free sphingoid bases are essential secondary mediators, mediating various cellular processes, including growth, proliferation, DNA synthesis, and Cer biosynthesis [196]. These bases can readily traverse membranes, suggesting their potential involvement in stimulus-induced changes in membrane permeability. However, pinpointing the exact signaling functions of sphingoid bases is likely challenging due to their various signals and immense interaction with numerous cellular molecules, such as CHOL, phospholipids, and proteins [197,198,199,200]. It is necessary to highlight that dietary SLs have a proportional direct impact on their detected levels in cellular membranes and tissues [201]. In addition, a number of compounds, such as fumonisin (FUM) mycotoxins and Alternaria toxins [202], share structural similarities with free sphingoid bases, enabling them to interfere with sphingolipid metabolism and alter cellular signaling.

#### 2.3.2. Ceramide

Cer is a non-bilayer-forming lipid characterized by its composition of a sphingosine base and a single acyl chain bonded to an amide group, thus lacking a distinct head group, illustrating its hydrophobic nature. The simple structure of Cer bears a resemblance to DAG. Cer serves as one of the simplest SLs and functions as the core building block for more complex SLs [198,203,204], which have larger molecular dimensions. Cer can be synthesized through multiple pathways: (1) de novo synthesis from SA substrate in the ER (a process catalyzed by *N*-acyl transferase/ceramide synthase (CerS)) and dihydroceramide desaturase [194]; (2) in vivo turnover of complex SL found in plasma membranes and lysosomes catalyzed by enzymes such as sphingomyelinase (SMase) or non-lysosomal glucosylceramidase) [205]; and (3) the salvage pathway in lysosomes that involves the re-acylation of SO to produce Cer [206].

The key enzyme responsible for de novo Cer generation is CerS, a family of six integral membrane proteins (CerS1–6) located in the ER of mammalian cells and regulated by corresponding six genes situated at distinct chromosomes [207]. The expression of these protein isoforms varies among different tissues [208], leading to variations in Cer proportion and acyl chain lengths. The CerS enzyme is responsible for the formation of dihydroceramide (DCer), which is an intermediate in Cer synthesis. In this step, DCer is formed by acylating a fatty acid to SA, followed by a desaturation reaction catalyzed by DCer desaturase to produce Cer. A decade ago, DCers were commonly considered to be quiescent intermediate metabolites, but recent research has unveiled their distinct functions compared to Cer [209]. Though de novo Cer production takes place in the ER [210], it has been suggested that long-chain bases are acylated in hepatic mitochondria. However, under specific events such as type 2 diabetes and FUM exposure, 1-deoxy-Cer and 1-deoxy-DCer are generated [211,212].

Cer plays a critical role in cellular signaling, regulating cell growth and apoptosis depending on the length of its acyl chain. Specific Cer species, like C16-Cer, have been proposed to be associated with apoptosis rates [213], while C18-Cer has been linked to growth arrest and a proportional decrease during cancer progression [214]. In addition, the ratio between C16 and C24:0/C24:1 Cer species has been identified as a factor in signaling induction, with C16 inducing apoptotic effects and C24:0/C24:1 exhibiting protective effects [215]. Therefore, alterations in the length of the Cer chain may potentially modify signaling, resulting in diverse metabolic effects. Recent review articles have highlighted the connections between Cer acyl chain length and cognitive functions [216] and intracellular lipid regulation [208]. Cer is also known for its ability to cluster apart from membranes, forming ceramide-rich domains with gel-phase properties. These domains are believed to act as platforms for protein–lipid interactions, selectively recruiting or excluding certain membrane components from small transit rafts. Cer-rich domains cluster these components in a stable manner, impeding their in-plane diffusion [217]. Therefore, the high hydrophobicity and complex polymorphic phase behavior of Cer [218] make Cer an important unit in lipid raft formation.

#### 2.3.3. Complex Sphingolipids

In mammals, complex SLs are present in two isomers: phosphosphingolipids (PSLs) and GSLs. Complex SL consists of Cer bonded to complex phosphoryl or carbohydrate moieties, located either in the lumen or at the cytosolic surface of the Golgi apparatus. The transport of Cer between the ER and Golgi organelles is regulated through vesicular and non-vesicular mechanisms, which involve Cer transfer protein [219,220]. This process is primarily coupled by complex SL migrations across membrane leaflets [221] and acts as a rate-limiting factor in the production of complex SLs.

##### Phosphosphingolipids

In the realm of PSLs, Cer binds to a phosphate group with a polar head group, forming a polar head group mainly composed of choline, ethanolamine, or glycerol. This structural distinction sets PSLs apart from PC in that they not only act as hydrogen bond acceptors but also as hydrogen bond donors. The PSL class includes various subtypes, such as Cer-1-phosphate (Cer1P), DCer-1-phosphate (Dcer1P), Cer phosphoethanolamines (CerPE), sphingomyelins (SM), dihydrosphingomyelins, and LysoPSLs (lacking an attached fatty acyl chain). Among PSLs, SM stands out as the most studied and highlighted class of PSLs in cellular membranes. This review primarily focuses on SM, omitting detailed discussions of other PSLs. However, Cer1P is the simplest PSL with its structure involving the esterification of Cer with the phosphate group. Cer1P serves crucial roles in the regulation of eicosanoids by activating the PLA2 enzyme [198,222].

##### Sphingomyelin

SM, also referred to as Cer-1-PC, is primarily of animal origin and constitutes a major fraction of SLs in the plasma membrane, accounting for approximately 15% of cerebral lipids [64]. SM is essential for the transmission of nerve impulses. It was initially isolated and described by Thudichum in 1884 [68]. SM is composed of Cer linked to a phosphocholine group [223], a process catalyzed by sphingomyelin synthase (SMS) [224]. Therefore, the overall configuration of SM closely resembles that of PC. SMS is comprised of multiple isomers, including SMS1 and SMS2, each with distinct active sites, with SMS1 situated in the lumen of the Golgi apparatus and SMS2 located on the plasma membrane [225]. SMS is not solely involved in SM production; it also modulates the generation of DAG during de novo synthesis, occurring simultaneously with SM synthesis. SM can also be produced from LysoSM through fatty acid acylation or the straightforward transmission of phosphocholine to Cer [226]. However, the specific enzymes involved in the latter event have yet to be identified.

Similar to PC, SM is primarily located in the outer leaflet of membranes, but it has also been detected in the nuclear envelope membrane [227], mitochondria [228], and liver chromatin [229]. Vesicular transport is the mechanism that facilitates the migration of SM from the Golgi apparatus to the plasma membrane [230], where possible degradation by sphingomyelinase (SMase) may occur, resulting in the generation of Cer. Remarkably, SMS2 catalyzes a contrasting mechanism for SM synthesis in the plasma membrane [224]. Elevated activity of SMase in the plasma has been associated with metabolic dysfunctions and diseases [231]. However, intracellular levels of SM are not exclusively determined by SMS and SMase activities but are also influenced by the dietary uptake of SM. A review by Yang and Chen [232] delves into potential aspects of SM utilization as a dietary supplement.

SM stands apart from PC, despite sharing the same PC head group. Its distinctive characteristics result from a higher proportion of saturated acyl chains and enhanced intermolecular hydrogen bonding capabilities. Unlike PC, SM serves not only as a hydrogen bond acceptor but also as a hydrogen bond donor. Consequently, SM is capable of being involved in various cellular signaling processes, encompassing functions related to proliferation, migration, and apoptosis [233,234]. Previous studies have elucidated how SO and LysoSLs can modulate protein kinase activities [235,236]. Furthermore, both Cer and SM play a role in modulating the uptake of cholesterol esters from high-density lipoprotein (HDL) particles by the target cells [237]. SM also plays a major role in the formation of lipid rafts, engaging in interaction with CHOL to form membrane microdomains [238,239,240], wherein roughly 70% of the cellular total SM is concentrated [241]. This favorable interaction between SM and CHOL can be attributed to the specific attributes of SM molecules, including their elongated saturated chains and reactivity properties (hydrogen donor and acceptor).

##### Glycosphingolipids

This lipid class closely resembles SM due to their shared origin from Cer. It is commonly referred to as GSL as it distinguishes itself from SM by replacing the complex phosphoryl group with a carbohydrate moiety. GSLs are largely derived from glucose moiety, resulting in the formation of glucosylceramide (GlcCer). In addition, GSLs can also be synthesized from a galactose moiety under the activity of galactosyltransferase, leading to galactosylceramide (GalCer) formation. It is essential to highlight that GSLs vary in their carbohydrate acylation locations. GlcCer is primarily produced at the cytoplasmic surface of the Golgi apparatus, whereas GalCers is made on the luminal side of the ER and is subsequently transported to the Golgi apparatus for further structural modifications to generate various GSLs [242,243]. Within cellular membranes, GSLs are believed to exhibit a preference for partitioning into lipid rafts and are involved in communication with the surrounding environment.

Hundreds of complex GSL structures are currently identified in biological systems, the vast majority of which are gangliosides, which are primarily composed of sialic acid and oligosaccharides [244]. Due to the intricate nature of this lipid class, in-depth classification and discussion have been deliberately avoided. However, two of the simplest GSL structures are glucosylceramide (GlcCer) and GalCer, often referred to as ‘cerebrosides’, featuring either a glucose moiety or a galactose moiety, respectively. The crucial translocation of GlcCer to the luminal leaflet of the Golgi apparatus is an essential step for its subsequent conversion into LacCer, an irreversible pathway involving the addition of a galactose molecule. In addition to these, there exist other GSL complexes such as sulfatides (containing sulfate) and globosides (featuring two or more sugar moieties, typically D-glucose, D-galactose, or N-acetyl-D-galactosamine), which have been identified as GSL derivatives in cellular contexts [245]. For the sake of simplification, scientists have categorized GSLs into two main groups: (1) neutral GSLs, which are characterized by glycosyl groups devoid of acids and remaining unsubstituted, and (2) acidic or amphoteric GSLs, whose glycosyl groups contain one or more sialic acids or a sulfate or phosphate group [246,247].

GlcCer and GalCer function as precursors for numerous complex GSLs, some of which possess additional carbohydrate groups numbering as high as 20 [248]. The addition of these carbohydrate moieties takes place in the Golgi luminal leaflet following the flip-flop translocation of simple GSLs. Majorly, GSLs serve two distinct functions [249]. Firstly, they act as cell receptors to their binding ligands, thereby acting as antigens while facilitating cell adhesion. Secondly, they function as signaling modulators by interacting with other membrane constituents, particularly growth factor receptors. Thus, GSLs play an essential role in immune-cell functions, with a large number of GSL molecules serving as tumor-associated antigens [250,251,252,253].

## 3. Fatty Acids of Biomembranes

Within the context of biomembranes, the matrix comprises an array of complex molecules, with fatty acids serving as fundamental building blocks. Fatty acids exist typically in two forms: saturated and unsaturated monocarboxylic acids, whereas both are characterized by a terminal carboxyl (-COOH) group and a terminal methyl (-CH_3_) group designated as carbon 1 (Δ) and omega (ω or n), respectively. Over the past century, numerous nomenclature systems have been proposed, including trivial, systematic, ∆^x^, n − x, and lipid numbers [22,23]. The trivial nomenclature, though prevalent, lacks systematic patterns. In contrast, the systematic nomenclature adheres to a more regular and structured approach, based on the nomenclature of parent hydrocarbons. It involves adding the suffix “oic” to the hydrocarbon name after removing the terminal “e”. This nomenclature also encompasses the identification of the position of the first double bond from the (n), with the series of fatty acids being named accordingly (e.g., n-3, n-6, n-7, and n-9 series). These distinctions among n-fatty acids lead to variations in their properties, consequently influencing the structure and function of biomembranes [254].

Concerning complex lipids, phospholipids, and SLs addressed in this review, fatty acids play a central role as their primary constituents. Therefore, it is essential to provide a concise overview of their biosynthesis, incorporation into complex molecules, and their biological functions in mammals.

### 3.1. Synthesis of Fatty Acids

Fatty acids can either be derived from the diet or biosynthesized within the cytosol and ER through an indigenous pathway known as de novo fatty acid synthesis. This synthesis is a complex process influenced by several determinants, including species, transcription genes, dietary composition, age, gender, stored lipids, and both endogenous (metabolic and interactive molecules) and exogenous (environmental) factors. A multitude of genes regulate the synthesis of fatty acids, which can vary among different species. In eukaryotic organisms, nearly 5% of the overall genes are responsible for a significant proportion of lipid structures [255]. Remarkably, the liver X factor (LXR) contributes to the regulation and synthesis of saturated, mono-, and polyunsaturated fatty acids by targeting their transcriptional genes [256]. It also indirectly influences encoding factors involved in lipogenesis, such as sterol regulatory element-binding protein 1c (SREBP1c) [257], peroxisome proliferator-activated receptor gamma (PPAR-γ) [258], and carbohydrate response element-binding protein (ChREBP) [259].

Numerous organisms can produce a wide variety of fatty acids, but only a limited number of molecular structures are synthesized in significant quantities at the natural physiological rate [260,261]. Generally, the synthesis activity of fatty acids is relatively low in normal adult cells, with the exception of certain tissues, including the brain, liver, adipose, and lungs [262,263]. The liver, known as a lipogenic organ, is predominantly responsible for the de novo pathway, although the white adipose tissue (which consists of lipogenic cells) and mammary glands in animals and humans also possess the capability to produce fatty acids through de novo lipogenesis [264,265,266,267]. Under conditions of energy equilibrium, the liver takes up a remarkable proportion (30–50%) of free fatty acids continually absorbed from the diet. These assimilated lipids are either directly incorporated into phospholipids and triglycerides (TAGs) or subjected to modifications (including elongation and/or desaturation) to produce new/modified fatty acids.

**Figure 5:**
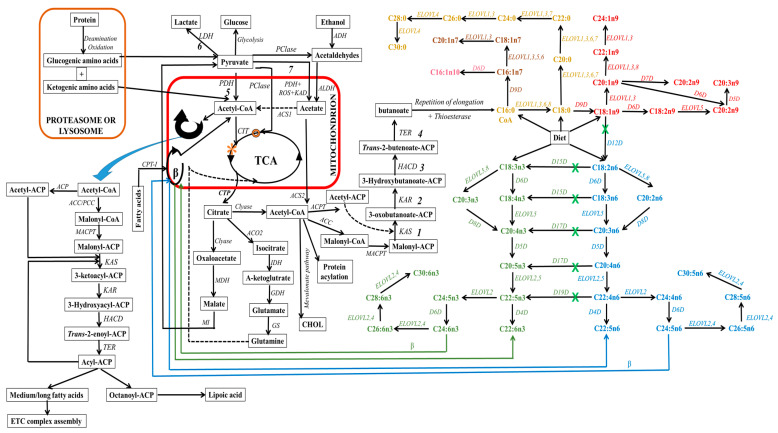
Schematic depiction of eukaryotic fatty acid biosynthesis and modification pathways, with emphasis on enzyme involvement (in italics). In this schematic, mitochondria are denoted by the red box, while proteasome/ribosome components are indicated by the orange box. However, light orange text delineates the elongation pathway for diverse saturated fatty acids. Within the diagram, both green and blue hues, accompanied by corresponding colored arrows, elucidating the discrete pathways for n3 and n6-fatty acid synthesis, respectively. On the other hand, text with color spectrum transitioning from pink to red designates the pathways for the synthesis of various monounsaturated fatty acids. The green “X” indicates the unattainability of this pathway in mammals, particularly higher eukaryotes, owing to the absence of a specific enzyme. ELOVL8 is a fish-specific elongase. Abbreviations: 1, condensation; 2, reduction; 3, dehydrogenation; 4, reduction; 5, aerobic conditions; 6, hypoxia or anaerobic conditions; 7, aerobic conditions; ACC, acetyl-CoA carboxylase; ACP, acyl carrier protein; ACPT, acyl carrier protein transacylase; ACS1, acetyl-CoA synthetases-1; ACS2, acetyl-CoA synthetases-2; ADH, alcohol dehydrogenase; ALDH, aldehyde dehydrogenase; ACO2, aconitase; β, beta oxidation; CHOL, cholesterol; CIT, citrate synthase; Clyase, citrate lyase; CPT-I, carnitine-palmitoyl transferase-I; CTP, citrate transporter protein, EAR, enoyl-ACP reductase; ETC, electron transport chain; GDH, glutamate dehydrogenase; GS, glutamine synthetase; HACD, β-hydroxyacyl-ACP dehydrase; IDH, isocitrate dehydrogenase; KAD, keto acid dehydrogenase; KAR, β-ketoacyl-ACP reductase; KAS, β-ketoacyl-ACP synthetase; MACPT, malonyl-CoA:ACP transacylase; MDH, malate dehydrogenase, MI, malic enzyme, PCase, pyruvate carboxylase; PCC, propionyl-CoA carboxylase; PDH, pyruvate dehydrogenase; ROS, reactive oxygen species; TCA, tricarboxylic acid cycle; TER, trans-enoyl-ACP reductase; O, oxaloacetate; *, citric acid).

Lipogenic cells can synthesize fatty acids endogenously from non-fat molecules, such as glucose or amino acids (see Figure 5). In this process, pyruvate, a metabolite of glucose, enters the mitochondria, where it undergoes oxidative decarboxylation through the pyruvate dehydrogenase complex to form acetyl-CoA. Within the mitochondria, acetyl-CoA can also be derived from the degradation of ethanol, proteins (deamination and oxidation), and fatty acids undergoing β-oxidation. Subsequently, these produced acetyl-CoA enter the tricarboxylic acid cycle (TCA, citrate cycle, or Szent–Györgyi–Krebs cycle). This event is very crucial since mitochondrial acetyl-CoA molecules are not permeable to mitochondrial membranes. Thus, they are initially located within the mitochondria, whereby they endure a condensation reaction with oxaloacetate to form citrate, a process catalyzed by citrate synthase during TCA [268,269]. The citrate molecule is then expelled from the TCA cycle to the inner mitochondrial membrane, and subsequently to the cytosol. This citrate transporting event requires a dicarboxylate antiporter solute carrier family 25 (SLC25A1) [270].

In the cytosol, citrate can undergo distinct metabolic pathways to generate various metabolites (see Figure 5). For instance, ATP-citrate lyase enzymatically cleaves citrate into acetyl-CoA, which leads to the carboxylation of acetyl-CoA and the formation of malonyl-CoA. Within cellular cytoplasm, acetyl-CoA (an active form of acetate) and malonyl-CoA serve as the primary substrates that initiate the process of carbon chain elongation. It is worth noting that propionyl and short-branched acyl units for priming can also be utilized in specific cases. This occurs, for instance, when adipose tissue contains monomethyl-branched fatty acids [271]. Additionally, the elongation process during the synthesis of branched fatty acids in specific glands requires the incorporation of methylmalonyl units [272].

It is imperative to emphasize the critical role played by the acyl carrier protein (ACP), which binds to acetyl-CoA and malonyl-CoA, a process catalyzed by malonyl-CoA:ACP transacylase. This integration event facilitates cytosolic elongation in higher eukaryotes by sequentially transferring these substrates from one enzyme/enzyme domain to another throughout sequential biosynthetic cycles. This active participation of ACP is vital for fatty acid biosynthesis and the functions of fatty acid synthase (FAS), which is a multi-enzyme system regulated by the encoded FASN gene. This cytosolic de novo pathway comprises a series of reactions catalyzed by acetyl-CoA carboxylase (ACC) and FAS, which serve as rate-limiting enzymes [273,274,275]. These reactions include ATP-dependent carboxylation of acetyl-CoA to form malonyl-CoA, Claisen condensation to extend malonyl-ACP and form 3-oxobutanoate, ketoreduction to yield 3-hydroxybutanoate, dehydration to yield butenoate, enoyl reduction to yield butanoate, and repeating elongation reactions (see Figure 5). This process results in the elongation of carbon chains up to the length of C16 or C18 in the cytosol [276,277,278].

ACC, the rate-limiting enzyme in the de novo pathway, facilitates the irreversible decarboxylation of acetyl-CoA through the addition of CO_2_ to produce malonyl-CoA. The resulting malonyl-CoA attaches to ACP and also serves as a two-carbon donor within a cyclic sequence of reactions facilitated by FAS, leading to the generation of a variety of fatty acid species. The end products (acyl-ACP) of cytosolic de novo synthesis are primarily palmitic acids (C16:0), with lower extents of myristic (C14:0) or stearic (C18:0) acids originating from acetyl-CoA. The determination of chain length during cytosolic de novo biosynthesis involves three enzymes: acyltransferases, ketosynthases, and thioesterases [279]. It is essential to highlight that there are two ACC isoforms: ACC1 (also known as ACCα), which is highly expressed in adipose and hepatic tissues, and ACC2 (also known as ACCβ), which is highly expressed in the heart and skeletal muscles [280]. The mechanism for the conversion of acetyl-CoA to malonyl-CoA by ACC is suggested to differ depending on the ACC type due to their different expression locations [281]. ACC1 is a cytosolic enzyme, whereas ACC2 is located in the outer mitochondrial membrane. On the other hand, FAS enzymes are exclusively cytosolic and can catalyze the formation of C16:0 from acetyl-ACP (substrate) and malonyl-ACP (2-carbon donor) [274,275]. FAS comprises numerous large-multifunctional protein domains (type I FAS) in eukaryotic and specific bacteria, while a monofunctional polypeptide domain is present in the case of major bacteria (type II FAS) [279,282,283,284,285].

In animals, the FAS pathway undergoes termination through a process involving a thioesterase, resulting in the liberation of the free fatty acid as the final product. The termination of the repeating elongation process exhibits the greatest degree of variation in fatty acid biosynthesis. Nevertheless, the conversion of the cytosolic elongated product to the CoA-ester is vital for further biosynthetic pathways that generate new fatty acid structures.

#### 3.1.1. Elongating Fatty Acids through Non-Cytosolic Mechanisms

Generally, the incorporation of the product into lipid structures or its involvement in subsequent elongation and/or desaturation processes is contingent upon the specific requirements of the organism at a given time. Further elongation pathways are not exclusive to fatty acids derived from de novo fatty acid synthesis in the cytosol; they also act on fatty acids derived from the diet, further extending and/or desaturating them to produce longer saturated, monounsaturated, or polyunsaturated fatty acids that are vital for all biomembranes. Following the production of palmitic acid in the cytosol, further modifications of this fatty acid may occur within cell organelles. In mammals, these modifications involve elongation (chains of 18 carbons or longer) and/or desaturation (formation of monenoic/monounsaturated fatty acids).

The ER and mitochondria regulate the modification (elongation) of fatty acids [278] in order to provide sufficient specific signals and functions. Already existing saturated fatty acids are elongated by the sequential addition of two carbon atoms, resulting in the formation of new fatty acids [286]. The principal fatty acid elongation pathway at the cytosolic side of the ER involves a series of four independent reactions: (1) condensation, (2) reduction, (3) dehydration, and (4) a final reduction step [287]. Major enzymes involved in the elongation process include 3-ketoacyl-CoA synthases (elongase enzymes or ELOVLs for reaction 1), 3-ketoacyl-CoA reductase (for reaction 2), hydroxyacyl-CoA dehydratase (for reaction 3), and *trans*-2,3-enoyl-CoA reductase (for reaction 4). The ELOVL family, sometimes referred to as type III FAS, serves as the rate-limiting enzyme family in the elongation pathway. This family consists of seven subtypes in mice, rats, and humans, and their regulation is governed by ELOVL-encoded genes.

ELOVLs catalyze the condensation of acyl-CoA and malonyl-CoA, which is responsible for elongating fatty acids and determining their carbon chain length, thereby influencing the cell’s fatty acid composition and signaling. ELOVLs exhibit variation in substrate specificity, tissue distribution, and regulation [288]. Based on their final products (see Figure 5), ELOVLs are classified into groups: ELVOLs that elongate saturated and monounsaturated fatty acids (ELOVL1, 3, 6, and 7), ELVOLs that produce very long-chain polyunsaturated fatty acids (ELOVL2 and 4), ELVOL5, which acts on a wide range of substrates with carbon chains ranging from 16 and 22, and ELOVL8, which acts on a wide range of substrates with carbon chains ranging from 16 to 20. ELOVL8 is a distinct subtype that has been recently discovered but is believed to be specific to fish [289]. It is widely acknowledged that these genes are primarily regulated at the transcriptional level; however, additional regulatory mechanisms may exist, including allosteric inhibition. In mammals, ELOVL4 is the sole enzyme capable of catalyzing the formation of fatty acids with more than 26 carbons [290]. These polyunsaturated fatty acids with more than 28 carbon atoms are primarily found in the retina, brain [291,292,293,294], testis [295,296], spermatozoa [297], epidermis [298], meibomian gland [299,300], and Vernix caseosa [301].

An additional pathway for elongating fatty acids occurs in non-cytosolic fatty acid synthesis, especially in the mitochondria. Both animals and yeasts possess mitochondria that contain FAS II enzymes (mtFAS II), which appear to interact with ACP-linked molecules [302,303]. It should be noted that the ACC enzyme has not been identified in the mitochondria of most mammalian species, including humans. However, a recent isoform of ACC1 has been identified in the mitochondria of mice [304]. Thus, isoforms of ACC1, and potentially mitochondrial propionyl-CoA carboxylase [305], are believed to regulate the decarboxylation of acetyl-CoA to yield malonyl-CoA within mammalian mitochondria. Nevertheless, these reactions occur at a limited rate, suggesting that imported malonate may play a role in mitochondrial fatty acid synthesis.

Mitochondrial fatty acid elongation relies on nicotinamide adenine dinucleotide phosphate (NADPH)-dependent enoyl-ACP reductase, with acetyl-ACP and acyl-ACP serving as substrates [306]. This pathway appears to be energetically unfavorable and represents a minor pathway in eukaryotes [278], primarily contributing to the generation of fatty acids used in the lipogenesis of mitochondrial membranes and cellular respiration. Unlike animals, where thioesterase-mediated termination is involved, mitochondrial termination entails channeling the mitochondrial acyl-ACP into the lipid biosynthetic pathway [307]. The primary generated product of mtFAS II activity is an octanoyl chain, which serves as a substrate for lipoic acid synthesis—a vitamin that acts as a scavenger for free radicals [308,309,310] and enhances energy metabolism as a cofactor [311]. Although this pathway can also generate medium and long fatty acids [312], their exact biological roles remain uncertain. However, studies by Nowinski et al. [264] and Angerer et al. [313] have suggested that these mitochondrial long-chain fatty acids are involved in the electron transport chain (ETC) complex assembly.

Furthermore, a similar pathway for fatty acid elongation is proximal fatty acid elongation, which is characterized by reversible β-oxidation. In this pathway, acetyl-CoA acts as the carbon donor, and peroxisomal *trans*-2-enoyl-CoA reductase substitutes acyl-CoA dehydrogenase to facilitate a thermodynamically favorable reaction [314]. It is important to emphasize that CoA is implicated in the fatty acid catabolism of the reversible pathway, while ACP plays a role in mitochondrial fatty acid elongation. However, the precise functions of fatty acid elongation within peroxisomes remain insufficiently characterized from a scientific standpoint. In practice, the extent of elongation is typically assessed using the elongase estimated index, which is determined by the ratio of C16:0 to C18:0.

#### 3.1.2. Desaturation of Fatty Acids

Within the context of de novo fatty acid synthesis, a process characterized by the removal of two hydrogen atoms to create a double bond often intersects with the fatty acid elongation pathway. This synergy ensures the production of long-chain and very long-chain unsaturated fatty acids, both mono- and polyunsaturated. Notably, the enzymes responsible for fatty acid desaturation, known as fatty acid desaturases, are ubiquitous across all domains of life with the exception of archaea, where they are notably absent [315]. However, it is of significant importance to underscore that the synthesis of polyunsaturated fatty acids can also occur independently of the classical series of desaturase and elongase enzymes. Several studies, including those conducted by Smith and Tsai [316], Kaulmann and Hertweck [317], Napier [318], and Metz et al. [319], have extensively documented an alternative pathway for the biosynthesis of long-chain polyunsaturated fatty acids in both prokaryotes and lower eukaryotes. This alternative route relies on semi-fatty acid synthesis systems, specifically known as polyketide synthases (PKS). PKS employs the same four fundamental reactions as FAS. However, the PKS cycle is frequently condensed, resulting in the formation of highly modified carbon chains featuring numerous keto and hydroxy groups, along with trans-configured double bonds that exhibit various functional roles [320,321].

Among the plethora of desaturase families found in different species, researchers have categorized them into three distinct types, as described by Cerone and Smith [322]. The first family, acyl-acyl carrier protein desaturase, is exclusive to plastids of higher plants. The second family, acyl-lipid desaturases, is found in the ER membranes of higher plants and cyanobacteria. The third family is the family of acyl-CoA desaturases, which can be present in both eukaryotes and prokaryotes, and these enzymes use a cytochrome b5-like system during oxygen reactions [323]. Within the context of this review, with a primary emphasis on mammals, the discussion will be specifically on the acyl-CoA desaturase families. In mammals, a critical in vivo biosynthetic route for the production of long-chain polyunsaturated fatty acids is known as the ‘Sprecher pathway’ [324]. This pathway relies on two fatty acid desaturase enzymes, two ELOVLs, and a peroxisomal β-oxidation process.

The desaturase pathway encompasses diverse enzyme families, such as stearoyl-CoA desaturase (SCD) [325] and fatty acid desaturase enzymes (FADS) [326]. Each of these enzymes acts on distinct substrates. For example, FADS primarily targets polyunsaturated substrates, while SCD predominantly acts on saturated substrates. SCD, also referred to as delta-9 desaturase (∆9-desaturase, or D9D), is an ER enzyme that catalyzes the formation of monounsaturated fatty acids, including palmitoleic (C16:1 n7) and oleic (C18:1 n9) acids, from saturated fatty acids, such as palmitic and stearic acids, respectively. These enzymes exhibit varying specificities and can work on a range of fatty acids with different carbon chain lengths, from 16 to 26 carbon chains. This variation hinges on the specific isoform present, with some species harboring multiple homologues of D9D. For instance, two homologues (D9D-1 and D9D-2) have been identified in marine copepods [327].

Basically, within the D9D reaction, a double bond is introduced at the 9th position from the carboxyl group of the fatty acid. In addition, a multitude of desaturase enzymes present in plants, bacteria, and insects perform the initial double bond introduction on saturated fatty acids at various positions, including ∆3, ∆5, ∆7, and ∆11 [328,329,330,331,332]. The activity of D9D is modulated by dietary fatty acid intake and is subject to up-regulation by the expressions of SCD gene isoforms [333]. Since Bloch’s 1960 pioneering discovery of SCD [315], a plethora of gene isoforms have come to light. In mice, the SCD exhibits four distinct isoforms (SCD-1–4) [334], whereas in humans, only two isoforms (SCD-1 and SCD-5) have been identified [335]. These gene isoforms can vary in substrate preference, ∆ position, and double bond conformation [336]. SCD-1 is the most studied isoform among them [325]. The importance of the SCD pathway is underscored by its involvement in cellular stress, lipid metabolism, inflammation, and autoimmunity. Among the isoforms, SCD-1 is the most extensively studied and is associated with various physiological processes [337,338,339,340,341,342].

Fatty acids featuring a ∆9 double bond are eligible for elongation, but it is important to note that these fatty acids may also be derived from the diet. However, D9D activity is indirectly determined by assessing the ratio between C18:0 and oleic acid. Notably, the de novo elongation of oleic acid results in the formation of longer chains of monounsaturated fatty acids. Furthermore, oleic acid, in particular, may undergo a desaturation process often followed by elongation, where the double bond is introduced before the ∆9 position. Nevertheless, unlike in plants and a limited number of animal species, mammals lack the enzymatic capability to catalyze the introduction of the second double bond in oleic acid, particularly not after the Δ9 position.

Fatty acid desaturase genes (FADs) comprise a gene family responsible for encoding a variety of desaturase enzymes. These enzymes play a significant role in catalyzing the synthesis of polyunsaturated fatty acids by introducing multiple double bonds at positions within fatty acids. Among the genes involved in fatty acid desaturation, mammals have been identified with four distinct FADs [335]: (1) FAD-1, (2) FAD-2, (3) FAD-3, and (4) FAD-6. Each of these FAD types tends to have alternative transcriptions [343,344,345], which can express distinct desaturase activities at different ∆-positions. Generally, FAD enzymes can be categorized into FAD-1, responsible for generating omega-3 (n3) fatty acids; FAD-2, involved in generating omega-x (nx) fatty acids; and FAD-3, which contributes to the formation of omega-6 (n6) fatty acids. While little is known about the substrates of FAD-6 and their major roles have not yet been defined, it appears to be homologous to human FAD-2 [346,347,348] and likely plays a role in the synthesis of polyunsaturated fatty acids.

The transcription factor FAD-6 has been described to possess Δ4-, Δ5-, and Δ8-desaturation activities, with a notable impact on docosahexaenoic acid (C22:6 n3, or DHA) synthesis from n3-docosapentaenoic acid (C22:5 n3, or DPA-n3) in golden pompano fish [349]. On the other hand, Park et al. [345] detailed the existence of nine alternative transcriptions for FAD-3, potentially arising from splicing events. Initially identified through cloning efforts by Marquardt et al. [350], FAD-3 has been traditionally classified as a non-polyunsaturated desaturase, primarily due to its limited substrates, namely, vaccenic acid (C18:1 trans-11) and sphingoid bases. In this regard, it should be noted that FAD-3 may facilitate the unanticipated ∆13-desaturation of trans-vaccenate [351]. These limited substrates give rise to the production of 11E,13Z-octadecadienoic acid [352] and 4E,14Z-sphingodienine [353], respectively.

In contrast to FAD-3, FAD-1 and FAD-2 are the principal contributors to the biosynthesis of polyunsaturated fatty acids in mammals. Nevertheless, mammals lack two crucial desaturases, namely delta-12 desaturase (∆12-desaturase, or D12D) and delta-15 desaturase (∆15-desaturase, or D15D) [354,355,356]. These enzymes are often referred to as “methyl-end desaturases” due to their ability to introduce a new double bond between an existing unsaturated bond and the methyl terminus (–CH_3_) of the fatty acid. Thus, mammals are de novo incapable of introducing a new double bond after the ∆9 position of oleic acid. Hence, mammals must obtain polyunsaturated fatty acids from their diet, leading to the concept of essential fatty acids. These essential fatty acids, such as linoleic acid (C18:2 n6, or LA) and α-linolenic acid (C18:3 n3, or ALA), cannot be endogenously synthesized by mammals and must be sourced from dietary intake [357,358,359]. Nonetheless, the enzyme activities of FAD-1 (∆5-desaturase, or D5D) and FAD-2 (∆6-desaturase, or D6D) have been identified in mammals. These enzymes are responsible for introducing double bonds at the ∆5 and ∆6 positions, respectively [360,361]. Notably, both D6D and D6D are highly expressed in the liver, with D6D exhibiting particularly high expression levels [362].

In mammals, dietary LA, ALA, and other unsaturated fatty acids, whether from the diet or synthesized endogenously, serve as substrates for the generation of polyunsaturated fatty acids characterized by an increased number of double bonds and longer carbon chains. The enzyme D6D plays a crucial role in the initial steps of synthesizing arachidonic acid (C20:4 n6, or AA), eicosapentaenoic acid (C20:5 n3, or EPA), and DHA by catalyzing the conversion of LA and ALA into γ-linolenic acid (C18:3 n6) and stearidonic acid (C18:4 n3), respectively [362]. However, the biosynthesis of AA and EPA, in particular, involves an additional desaturase enzyme, delta-5 desaturase (D5D), which utilizes the substrates dihomo-γ-linolenic acid (C20:3 n6, or DGLA) and eicosatetraenoic acid (C20:4 n3) to yield AA [360] and EPA [363], respectively. According to Vagner and Santigosa [364], the substrate affinities of D6D appear to exhibit contrasting and debatable characteristics. Consequently, further investigations are imperative to substantiate a definitive conclusion. It is essential to emphasize that the distinctive substrate affinities of D6D play a critical role in determining the ratio of n6- to n3-polyunsaturated fatty acids (referred to as n6/n3 or n6:n3). The role of FAD-6 in determining the ratio of n6/n3-polyunsaturated fatty acids and its influence on the metabolic flux of these fatty acids have been highlighted [365,366].

Remarkably, D6D and D5D are also involved in the synthesis of n9-polyunsaturated fatty acids, specifically, Mead acid (C20:3 n9), which is produced in the absence of LA and ALA [367], when only monounsaturated fatty acids are available as substrates. Elevated levels of Mead acid are matched with the proportional depletion of n6- and n3-polyunsaturated fatty acids and serve as a biomarker for LA and ALA deficiency in diets. For instance, the ratio between trienoic and tetraenoic acids (such as Mead acid:AA) functions as a biomarker for the lack of dietary essential fatty acids [368,369]. Park et al. [370] have identified two pathways for Mead acid production, involving D6D and D5D. These pathways vary in substrates of D6D, which can either utilize oleic acid or gondoic acid (C20:1 n9) to yield C18:2 n9 and C20:2 n9, respectively. D5D catalyzes the direct conversion of C20:2 n9 into Mead acid by introducing a double bond at the ∆5 position. Furthermore, authors have also reported the novel activity of ∆7-desaturase (D7D, regulated by FAD-1), leading to the formation of C20:2 n9 from C20:1 n9.

Notably, AA, EPA, and DHA are biologically essential fatty acids with enormous contributions to membrane phospholipids. In the Sprecher pathway, the primary fatty acid synthetic pathway in mammals, the biosynthesis of DHA from EPA is not direct but rather involves a series of reactions: (1) elongation to a 24-carbon chain, (2) desaturation via D6D, and (3) peroxisomal β-oxidation for chain shortening. However, the synthesis of n6-docosapentaenoic acid (C22:5 n6, or DPA-n6) and DHA in eukaryotes also occurs through ∆4-desaturase (D4D) pathways, depending on the species. In lower eukaryotes, marine vertebrates, and humans, D4D (encoded by the FAD-2 gene), which is expressed to a lesser extent, plays an essential role in introducing a double bond at position ∆4, directly producing DPA-n6 and DHA from docosatetraenoic acid (C22:4 n6, or adrenic acid) and DPA-n3 substrates, respectively [371,372,373,374]. It is noteworthy that this reaction bears resemblance to that facilitated by FAD-6 expression, suggesting the possibility of FAD-6 up-regulating D4D activities.

A further marked expression of FAD-2 identified within mammalian cells is ∆8-desaturase (D8D). This enzyme establishes an autonomous pathway distinct from D6D, introducing an additional double bond to substrates like ALA, LA, and their elongated polyunsaturated fatty acids, resulting in the formation of very long-chain polyunsaturated fatty acids [375,376]. For instance, the emergence of D8D was observed when DGLA and eicosatetraenoic acid were derived from eicosadienoic acid (C20:2 n6) and eicosatrienoic acid (C20:3 n3), respectively [377]. In a study conducted on mouse liver, D8D expressed low activity, which is primarily associated with n3-unsaturated fatty acids, such as eicosatetraenoic acid (C20:4 n3), EPA, DPA-n3, DHA, and nisinic acid (C24:6 n3) [375].

In general, more than a hundred FAD-2-related desaturase enzymes have been identified in various animal species, although they are notably absent in mammals. For instance, the ∆17 (17-desaturase, or D17D) and ∆19 (19-desaturase, or D19D) desaturases have been identified in lower animal families (excluding mammals) and algae [378,379]. These enzymes play an essential role in the production of EPA and docosapentaenoic acid-n3 (C22:5 n3, or DPA-n3) from AA and adrenic acid, respectively. The activities of enzymes involved in lipogenesis are subject to intricate regulation by a matrix of genes and exogenous factors. Notably, polyunsaturated fatty acids have been shown to inhibit the transcription of hepatocellular genes responsible for encoding lipogenic enzymes [380,381]. It is a common practice to assess desaturase enzyme activity indirectly by determining their coefficients based on the ratio of the fatty acids generated to the substrates utilized.

Desaturase enzymes exhibit a broader scope of activities beyond their involvement with fatty acid substrates incorporated into phospholipids, as they are also active in SLs. Ordinarily, SLs are characterized by their predominantly very long saturated or monounsaturated nature. The determination of fatty acid chain length within SLs is intricately governed by the activities of the CerS type [382]. However, it is worth noting that polyunsaturated fatty acids show a slight accumulation in SLs within testes and spermatozoa [383,384] as compared to phospholipids within the same tissues. The classification of desaturases responsible for introducing double bonds into Cer structures has been presented by Nachtschatt et al. [385]. This classification delineates three distinct categories of desaturases: (1) α-hydroxylases [386], (2) D4D and C4-hydroxylases [387], and (3) D8D [388]. These desaturases play a pivotal role in diversifying the composition of SLs, particularly in terms of the introduction of double bonds, which contribute to the functional and structural heterogeneity of these important lipid molecules.

### 3.2. Incorporation of Fatty Acids into Lipids of Biomembranes

**Figure 6:**
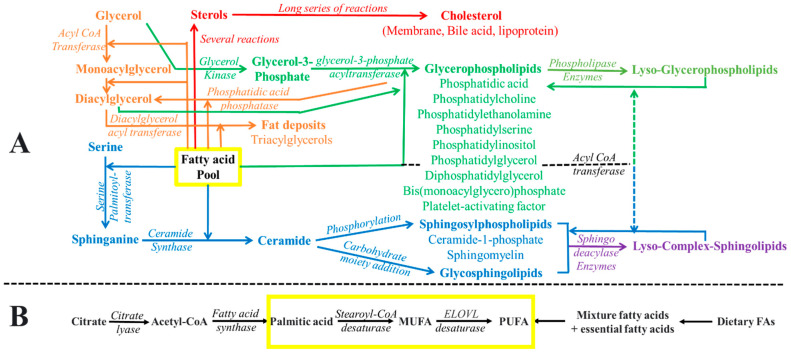
Schematic representation of (**A**) fatty acids incorporated into various lipids and (**B**) the origins of fatty acid pool formation (the de novo pathway and dietary sources). The depiction employs color coding to symbolize distinct metabolic pathways. The yellow box designates the fatty acid pool, signifying its integration into diverse membrane lipids. The brown shade denotes processes related to non-membrane and non-polar lipid formation. The red color represents the metabolic routes responsible for sterol production. Additionally, the green color signifies the integration of fatty acids into various phospholipids, while the blue hue corresponds to the incorporation of fatty acids into diverse sphingolipids.

Fatty acid synthesis and their subsequent incorporation into biomembranes represent critical processes in the life of all organisms. The one exception to this rule is the archaea domain, which employs isoprenoids as membrane lipid side chains rather than fatty acids [389]. Understanding how fatty acids are incorporated into membrane lipids is of paramount importance. Fatty acids may become part of phospholipids either through acylation of glycerol-3-phosphate during the biosynthesis of phospholipids or through the action of lysophosphatidic acyltransferases and phospholipases that remodel the structure of pre-existing phospholipids [390,391] (which can be seen in Figure 6).

The vital nature of these processes is underscored by the coexistence of diverse fatty acid structures within complex biomembrane lipids [392]. This structural diversity arises from various factors, including the preferences of *sn*-positions for particular fatty acids, the substrate preferences of biosynthetic enzymes, and the dynamic lipid remodeling program. Notably, studies by Shindou et al. [393] and Coleman et al. [394] have elucidated the preferences of specific enzymes for distinct fatty acids. For instance, acyl-CoA synthetase long-chain family members 3 and 4 demonstrate preferences for AA and EPA, while 1-acylglycerol-3-phosphate O-acyltransferase-α prefers myristic acid, palmitic acid, and LA. Meanwhile, 1-acylglycerol-3-phosphate O-acyltransferase-β exhibits a preference for AA.

In the context of lipid remodeling, the replacement of fatty acids within existing phospholipids plays a central role. For instance, the incorporation of C20 fatty acids generally takes place post-de novo synthesis, necessitating the employment of the remodeling pathway [395]. Hence, this process involves the conversion of one distinct phospholipid into another [85], highlighting the importance of the Lands cycle in this process. The Lands cycle [396,397] is a central component of the remodeling process, enabling the attainment of specific structural configurations required for the generation of particular cellular signals. In this pathway, phospholipases initiate cleavage (deacylation) of fatty acids from phospholipids, resulting in the formation of free fatty acids and lysophosphatidate. On the other hand, acyltransferases function in a contrasting manner, acylating the requisite fatty acids into lysophosphatidate to generate phospholipids. This process is CoA-dependent, which is primarily due to the extensive utilization of CoA intermediates as substrates. Other remodeling pathways include the CoA-independent transacylation pathway and the direct transacylation pathway, which are specifically suited for highly unsaturated fatty acids such as AA, EPA, and DHA. In these pathways, transacylases catalyze the migration of fatty acids between molecular species of phospholipids. Remarkably, these remodeling pathways exhibit substantial variability across different tissues [395], as comprehensively reviewed [390].

Additionally, it is noteworthy that fatty acids are predominantly incorporated into SLs by the rate-limiting enzyme CerS. However, a remodeling mechanism can also come into play, modulating specific signaling and functional roles. For example, the work of Markham et al. [398] suggests that the accumulation of very long-chain fatty acids contributes to the formation of microdomains through increased hydrophobicity, membrane leaflet interdigitation, and the transition from a fluid to a gel phase. These structural transformations have significant implications for cellular function and signaling within membranes.

The movement of membrane phospholipids between bilayers involves the activities of various transmembrane lipid transporter proteins, namely, flippase, floppase, and scramblase. These proteins play distinct roles in the translocation of lipids and exhibit differential mechanisms of action. For instance, flippase facilitates the translocation of lipids from the exo-leaflet to the inner-leaflet, contrasting with the activity of floppase, which operates in the opposite direction, moving lipids from the inner-leaflet to the exo-leaflet. Notably, both flippase and floppase depend on ATP as an energy source for their functions. Conversely, scramblase functions as an ATP-independent transporter and orchestrates the bidirectional movement of lipids across membranes in a non-specific manner, allowing for the concurrent translocation of lipids from the inner to the outer leaflet and from the outer to the inner leaflet. Comprehensive insights into these membrane fatty acid transporters have been provided by the extensive reviews conducted by Samovski et al. [399] and Glatz et al. [16]. These reviews offer a detailed discussion of the mechanisms and significance of these proteins in lipid translocation processes within biological membranes.

### 3.3. Fatty Acid Composition in Biomembranes

The fatty acid composition of biomembranes plays a critical role in maintaining cellular homeostasis and ensuring proper functionality. It is worth noting that this composition is not static but can be modified/remodeled in response to homeoviscous adaptation. This process was initially described in algae [400] and has been later extended to non-homeothermic animals. In contrast, mammals are generally considered to have stable biophysical properties and lipid order within cellular membranes, but they can respond to changes in their dietary fatty acid compositions. Furthermore, certain mammalian cells potentially exhibit a lower degree of homeoviscous adaptation in response to the membrane curvature elastic stress [401]. The fact that there is not a single standardized composition for fatty acids in biomembranes should not be viewed as a flaw but rather as an indication of the intricate and dynamic nature of biological systems. The complexity and adaptability of fatty acid composition in biomembranes underscore the potential evolutionary advantage conferred by the ability to alter lipid structures.

The available literature, which will be discussed in subsequent sections, provides extensive data regarding the proportions of specific fatty acids in various lipid complexes within biomembranes. This section merely focuses on the major fatty acids identified in phospholipids and SLs, as these fractions are the central aspects of this review. Regardless, the length and degree of saturation of fatty acyl side chains in CHOL-esters can vary between different cells. This variation is primarily influenced by dietary factors and cell metabolism, and it has a direct impact on the stability and fluidity of the cellular membrane.

#### 3.3.1. Fatty Acid Profile of Phospholipids

Commonly, the acyl chains of phospholipids found at the *sn*-1 and *sn*-2 positions of the glycerol backbone are typically composed of a saturated fatty acid (such as C16:0 or C18:0) and an unsaturated fatty acid (with carbon chains of 18, 20, 22, or 24), respectively. Furthermore, these acyl chains exhibit variations in terms of their length, the number of double bonds, and the position of hydrogen atoms adjacent to these double bonds (whether in *cis* or *trans* configuration). It is important to note that *trans*-double bonds are relatively rare within mammalian membranes, while *cis*-double bonds are frequently abundant. In fact, the occurrence of *trans*-double bonds in mammals is far less frequent than in bacteria [402,403]. In some instances, identical acyl chains can be found at both *sn*-1 and *sn*-2 positions of glycerol [404,405]. It is worth highlighting that even when the number of carbons is the same, a slight mismatch may occur due to the *sn*-positions. This mismatch is a consequence of the *sn*-2 chain bending perpendicular to the membrane’s plane [406,407].

In the case of PA, the composition of the two fatty acids can vary across different cell types and constituents. Typically, dietary fatty acids and metabolic disorders play a substantial role in determining the composition of these acyl chains. PA is primarily composed of saturated and monounsaturated fatty acids, with carbon chains ranging from 14 to 24. Concerning PG, this class exhibits variations in the length and number of unsaturation of its fatty acids, depending on the cell type and the dietary fatty acids involved. In the PG of rat lungs, palmitic acid is the most abundant fatty acid, with unsaturated fatty acids constituting a smaller portion [408]. PG is unique among phospholipids due to its higher degree of unsaturation, with unsaturated fatty acids more likely to occupy the *sn*-1 position. Research by Xie et al. [409] suggests that the degree of unsaturation in PG may have varying effects on mouse keratinocyte proliferation.

Commonly, the fatty acid composition of PCs is typically determined post-synthesis, with various events, such as deacylation and reacylation, taking place during its remodeling [396,397]. These events, part of the Lands cycle, can also impact the composition of other phospholipids due to homeostatic mechanisms or metabolic implications [410]. Generally, PC exhibits variations in its fatty acid composition across species and cell types [411,412,413,414,415,416]. Saturated fatty acids, like palmitic or stearic acids, are typically abundant at the *sn*-1 position, while C18 unsaturated chains or longer polyunsaturated fatty acids like AA and DHA are more prevalent at the *sn*-2 position.

The fatty acid composition of PE is highly dependent on the particular cell, tissue, and physiological conditions. In contexts like chicken egg, rat liver, and brain, palmitic and stearic acids tend to occupy the *sn*-1 position, while AA, oleic, and DHA are more common at the *sn*-2 position [411,412,417]. Notably, PE in the erythrocyte membrane exhibits a greater tendency for the accumulation/recruitment of polyunsaturated fatty acids compared to PC [418]. Consequently, PE in this context contains more polyunsaturated fatty acids, primarily AA and DHA, at the *sn*-2 position, despite its diacyl structure bearing similarities to that of PC.

The composition of fatty acids in PS differs depending on the tissue type. Nonetheless, lipid remodeling and selective insertion of fatty acids are common processes that occur along the biosynthetic pathway. Therefore, the fatty acid composition of newly synthesized PS differs from that of its precursors, PE and PC. When PS was isolated from rat liver and cow brain and analyzed regiospecifically [412,414], it was observed that stearic acid was more abundant at the *sn*-1 position, while the proportion of palmitic acid was lower here. These data underscore the distinct fatty acid incorporation pattern exhibited by PS in comparison to PC and PE. Furthermore, the acylation of fatty acids at the *sn*-2 position has been shown to be tissue-specific, with high proportions of AA and DHA in the livers of rats and the brains of bovines, respectively. Similar findings regarding DHA in brain regions of mice and rats have been reported by Kim et al. [419] and Hamilton et al. [420]. However, stearic acid was the most abundant fatty acid at the *sn*-1 position in these cases. Remarkably, the incorporation of these unsaturated fatty acids into PS appears to be more extensive than in the case of PC, as revealed by these studies. The fatty acid composition of PS at its *sn*-positions plays a crucial role in determining its functional properties to varying degrees [93,421].

In mammalian cells, the composition of PI is characterized by the presence of stearic acid and AA in the *sn*-1 and *sn*-2 positions, respectively, as consistently demonstrated in various studies [411,412,422,423]. These two fatty acids collectively account for a substantial portion of PI acyls, typically ranging from 50% to 80% in the brain and liver. Additionally, oleic acid is frequently identified as the third most abundant fatty acid in the rat brain, while palmitic acid and DHA exhibit lower levels of acylation in this phospholipid. However, it is worth noting that Ulmann et al. [424] reported a distinct fatty acid composition in the rat brain, where oleic acid was the most prevalent, followed by stearic and palmitic acids. This variability in PI composition across studies may be attributed to a range of factors, including dietary influences and genetic variations. In general, PI exhibits a strong preference for AA in its acyl composition. Remodeling processes can lead to the deacylation of pre-existing PI, resulting in the formation of lyso-PI via the action of phospholipases. The incorporation of AA into lyso-PI is facilitated by lysophosphatidylinositol acyltransferase 1 [425].

The structure of fatty acids within CL greatly influences its shape and properties. Saturated chains tend to produce lamellar forms of CL, whereas unsaturated chains contribute to non-lamellar structures [38]. Thus, the distinctive fatty acid composition of CL is believed to play a critical role in its diverse biological functions across different cellular and subcellular membranes. CL remodeling is an essential process for CL formation, during which LA and DHA are primarily incorporated, influencing susceptibility to oxidation [426,427,428,429]. LA has been reported as the majority of CL fatty acids, often accounting for 80–90% of the composition [430]. Although C18 polyunsaturated fatty acids are the predominant constituents of CL, dietary fatty acid intake can influence its composition. Increased dietary supplementation levels of C20 polyunsaturated fatty acids and DHA have been shown to elevate their proportions in mammalian CL, as indicated by Berger et al. [431] and Wolff and Entressangles [432].

The structure of BMP exhibits variations in chain length and the degree of fatty acid unsaturation. Oleic acid is frequently identified as the most abundant fatty acid in BMP [126,132,433]. However, in certain cell types, polyunsaturated fatty acids such as LA and/or EPA and DHA have been reported to be highly accumulative [434,435,436,437]. In alveolar macrophages, for instance, Holbrook et al. [438] noted that oleic acid predominates along with either AA or DHA.

In intact tissues (e.g., neural tissue), palmitic acid typically represents the most abundant fatty acid within the alkyl group of PAF. Stearic and oleic acids may also be present but to a relatively lesser extent. The *sn*-2 position of PAF is often esterified with the acetyl group or other short-chain fatty acids [439]. However, in rat nervous tissue, *sn*-2 is predominantly occupied by unsaturated fatty acids, primarily AA and adrenic acid (C22:4n6) [440], indicating the profound impact of *sn*-2 composition on PAF activities. These longer n6-type fatty acids serve as potent precursors for eicosanoids.

#### 3.3.2. Fatty Acid Profile of Sphingolipids

In general, SLs exhibit a prevalent composition of very long-chain saturated and/or monounsaturated fatty acids, typically ranging from 18 to 34 carbon chains. Notably, some sphingolipid structures have been reported to contain odd-numbered fatty acid chains [201,441], suggesting a possible proportional elevation of these chains in the tissues of ruminants and coprophagous species. Despite the fact that the enzyme SPT utilizes palmitoyl-Coenzyme A to produce SA, it has the capability to utilize various other fatty-CoA substrates as well [442]. Sphingoid bases within SLs predominantly feature saturated aliphatic chains, with some instances of mono- and di-unsaturated chains. SO possesses a fixed *trans*-double bond between carbons 4 and 5. The chain length of sphingoid bases’ fatty acids typically falls within the range of 14 to 32 carbons [443].

Within the context of Cer, the variability in chain length is attributed to the diversity of CerS isoforms. In most scenarios, CerS5 and CerS6 predominantly provide chains with 14–18 carbons, CerS1, CerS4, and CerS2 contribute chains with 18–24 carbons, and CerS3 supplies chains with up to 34 carbons [382]. Cer features an acyl chain linked to an amide that is frequently saturated and considerably longer compared to those found in SLs containing sugar moieties [240]. The length of the Cer fatty acid is determined by ER elongase and desaturase complexes, rather than the Golgi apparatus, which produces complex SLs. Cer and SM are primarily acylated by palmitoleic and oleic acids, in addition to long and very long-chain fatty acids. In most organisms, including mammals, Cer can contain 2-hydroxylated fatty acids [444,445], which is a characteristic favoring interaction with SMS2 over SMS1 [446].

SM structures are characterized by long fatty acids with relatively high chain length inequality, leading to their interdigitation sensitivity. The cause of interdigitation is not fully understood but may involve the regulation and/or induction of proteins in response to specific fatty acids within SM under certain membrane constituents. In comparison to PC, SM typically contains more saturated and longer acyl chains, typically ranging from 16 to 24 carbons [415,447], and may include odd-numbered fatty acids [448]. The palmitic, stearic, behenic (C22:0), lignoceric (C24:0), and nervonic (C24:1 n9) acids are among the most frequently occurring fatty acids in SM [415,447,448,449,450]. Mammalian germ cells, in particular, are abundant in very long-chain fatty acids with up to 34 carbons [297,451], some of which may exist in a 2-hydroxylated form in certain mammals [452]. Double bonds are relatively rare in SM fatty acids, and, when present, they are often located at a distance from the membrane–water interface [447].

GSLs exhibit a greater variation in the chain length of their fatty acids compared to phospholipids, with some GSLs containing fatty acids with more than 16 carbons [453]. Although it is challenging to detect long and/or unsaturated fatty acids in GSL, a few studies have reported the presence of high proportions of long and very long fatty acids. For instance, stearic acid has been found to be more dominant than palmitic acid in the GSLs of the mouse brain [454]. Furthermore, the majority of GalCer in the brain is composed of very long fatty acids [455]. In gangliosides, lignoceric acid (C24:0) is the most abundant acyl chain, constituting up to 70% of total fatty acids in myelin [456], while stearic acid is the predominant component, making up 80% of total fatty acids in the human brain [457].

## 4. Fatty Acids and Cellular Functionality

The different chemical structures, physicochemical properties, and physiological functions of various fatty acids exhibit remarkable diversity. These distinctions have led to various categorizations, depending on the specific scientific focus. Traditionally, fatty acids have been recognized for their dual biological functions: as structural components of biomembranes and as sources of energy. However, contemporary perspectives on fatty acids have expanded to encompass their roles as bioactive molecules that contribute significantly to overall health. Pioneering work by Burr and Burr [458] and von Euler [459] underscored the diverse biological functions of fatty acids, particularly LA and ALA, highlighting their crucial roles in cellular signaling processes. This section focuses on fatty acids’ impact on membrane properties and avoids delving into their role in energy provision via β-oxidation (primarily relies on TAGs), which is a subject beyond the scope of this review.

### 4.1. Influence of Fatty Acids on Bilayer Properties

Fatty acids play an essential role as constituents in cellular membrane assembly, profoundly influencing the physicochemical attributes of these membranes. Biomembranes exhibit a discerning preference for incorporating specific fatty acids, particularly favoring long and very long polyunsaturated fatty acids that contribute to the formation of fluidic membranes. A study by Rodriguez-Estrada et al. [460] has associated long-chain lipid metabolites derived from LA and ALA with the preservation of membrane properties. Sensor proteins typically monitor and regulate the physicochemical properties of membranes [7,461,462]. Fatty acids exhibit variations in chain length and degree of unsaturation across different cellular contexts, catering to specialized functions. For instance, the study of Matveyenka et al. [463] has highlighted the correlation between the rate of insulin aggregation and the length and degree of the unsaturation of fatty acids. Therefore, maintaining equilibrium among various fatty acid species (saturated, monounsaturated, and polyunsaturated) within membranes holds a position of critical importance, as any deviations from this equilibrium could lead to modifications in membrane integrity and cellular metabolic signaling. Notably, Baccouch et al. [464], Hashimoto et al. [465], and Ibarguren et al. [466] have reported the effects of fatty acid composition on various aspects of membrane behavior, including fluidity/viscosity/rigidity, thickness, permeability, phase transitions, fusion, lateral pressure, flip-flop dynamics, and structural integrity.

The optimal functionality of membrane-bound enzymes, ion channels, and receptors is intrinsically linked to membrane rigidity and permeability, influencing the diffusion of biomolecules within the lipid bilayer. Incorporating higher proportions of long saturated fatty acids enhances membrane rigidity, as these fatty acids are notably stable, having higher melting points than unsaturated fatty acids [467], and tend to form close clusters [468], particularly at physiological temperatures, resulting in heightened membrane rigidity. In contrast, polyunsaturated fatty acids contribute to greater conformational flexibility in membranes, which is a trait dependent on their chain length, degree of unsaturation, and the positioning of hydrogen atoms relative to the double bond. The presence of unsaturated fatty acids introduces curves/bends (also known as “kinks”) in the hydrocarbon chains, leading to the formation of less densely packed lipids and more fluidic membranes [469]. However, the influence of polyunsaturated fatty acids on membrane fluidity may vary, particularly in different bilayer states [470]. For instance, EPA and DHA have demonstrated negligible effects on fluidity in liquid-crystalline states [471]. In contrast, within different membrane models, polyunsaturated fatty acids with four or more double bonds, specifically AA, EPA, n6-DPA, and DHA, have been reported to decrease membrane thickness [464,472,473,474], increase the tilt angle [472], and elevate membrane fluidity [475,476]. The degree of rigidity contributed by double bonds within fatty acids is contingent on various factors, including their conformation (*cis* or *trans*), the degree of unsaturation, and their relative positioning concerning the carboxyl group. According to Roach et al. [477], the membrane properties of fatty acids associated with *cis*-unsaturated fatty acids were markedly different from those of saturated and *trans*-unsaturated fatty acids. Typically, *cis*-isomers exhibit greater polarity and possess relatively higher boiling points compared to *trans*-isomers, although not as high as those of saturated fatty acids. Notably, the position of the double bond exerts a more substantial impact on boiling points than the number of double bonds [478]. *Cis*-double bonds have been identified as expanding the spatial area occupied by the fatty acid, thereby increasing membrane fluidity [479,480] and permeability. Moreover, phospholipids containing long-chain n3-fatty acids have shaped more disordered and flexible membrane structures compared to LysoPLs containing n6-fatty acids, underscoring the significant role of n3-fatty acids in shaping membrane integrity.

Unsaturated fatty acids influence biomembrane rigidity not only through their intrinsic molecular structure but also by modifying the proportions of other membrane constituents that contribute to rigidity. For instance, Schumann et al. [481] and Stillwell [482] investigated the role of polyunsaturated fatty acids in modulating raft characteristics, including size, stability, and distribution. Notably, polyunsaturated fatty acids have a reduced affinity for cholesterol (CHOL) compared to their saturated counterparts. Consequently, an increased incorporation of unsaturated fatty acids within biomembranes can result in loosely packed lipid structures. This, in turn, leads to the displacement of raft-associated proteins and the removal of SM and CHOL from lipid rafts. These alterations result in shifts in membrane rigidity and permeability [483,484,485,486,487,488,489,490,491,492]. Therefore, the degree of unsaturation plays a crucial role in modulating the flip-flop rate and the asymmetry/distribution of membranes. Cheng et al. [493] and Armstrong et al. [494] have substantiated a positive correlation between the trans-membrane flip-flop rate and the proportion of unsaturated fatty acids. In assessing membrane rigidity, ratios such as phospholipid/CHOL, PC/PE, and PC/SM (the unsaturation index) have been routinely employed.

It is rational to posit that membranes characterized by inadequate lipid packing correspondingly exhibit elevated permeability. Indeed, a considerable body of research has established a link between polyunsaturated fatty acids and heightened membrane permeability, reflecting the rate at which molecules traverse biomembranes. It has been observed that the incorporation of long and very long polyunsaturated fatty acids, such as ALA, AA, EPA, and DHA, increases the permeability and elasticity of biomembranes [464,469,495,496,497,498,499,500]. This augmentation facilitates the translocation of ions and molecules across the membrane. Mondal et al. [469] attribute the elevated membrane elasticity to the disruption of the robust hydrogen-bond network surrounding the charged lipid head groups by the polyunsaturated fatty acids. The effects of polyunsaturated fatty acids, particularly EPA and DHA, on elasticity (and consequently permeability) can exhibit variability within the same cell [501], depending on the presence of other cellular constituents. Notably, the presence of CHOL can modulate membrane properties [495]. DHA, in particular, elevates permeability more significantly than its precursor, ALA [502], underscoring the pivotal role of the degree of unsaturation and its elevated incorporation levels in the functions of vital cells. DHA promotes heightened hydration within the head group and inter-chain regions, thereby increasing permeability. This is primarily attributed to the elevated number of double bonds. As elucidated by Mitchell and Litman [503], the presence of water within the hydrocarbon bilayer region exhibits a positive correlation with the high number of double bonds.

In addition to its effects on membrane rigidity and permeability, the incorporation of DHA profoundly impacts various aspects of biomembrane dynamics. DHA remarkably alters lipid packing, phase behavior, curvature, elasticity, interleaflet lipid flip-flop rates, lipid phase separations, membrane fusion, and vesicle formation [464,494,504,505,506,507,508]. According to Mitchell and Litman [509], the packing-free volume increases in the following order: 16:0-18:1PC has a lower relative abundance than 16:0-22:6PC, which, in turn, has a lower relative abundance than 22:6-C22:6PC. The potential effect of highly unsaturated fatty acids on phospholipids, which are characterized by loose packing, appears to be closely linked with the promotion of membrane elasticity, vesicle exfoliation (the formation of “blebs”), fusion, and flip-flop processes. In this regard, fatty acids actively participate in the processes of cell fusion and modulate cell phase behavior. During cell fusion, two distinct lipid bilayers merge, resulting in the formation of a continuous bilayer structure and the mixing of the internal contents of the lipid bilayers. Consequently, alterations in fusion processes have been associated with curvature stress in membranes [510,511]. The impact of the degree of unsaturation on membrane fusion has been previously demonstrated by Ahkong et al. [512], Meers et al. [513], Ehringer et al. [502], and, more recently, Li et al. [504]. The configuration of double bonds plays a critical role in determining the extent of biomembrane fusion. According to Creutz [514], AA and oleic acid are particularly effective fusogens, whereas saturated and trans-unsaturated fatty acids exhibit negligible fusogenic activity.

In terms of phase behavior, different fatty acid compositions contribute to various phase transitions, including gel-to-fluid, hexagonal, and liquid phases. Short-chain saturated fatty acids and mono- and polyunsaturated fatty acids result in lower viscosities, contributing to the formation of more fluid membranes compared to long-chain saturated fatty acids [466]. The impact of unsaturated fatty acids is particularly evident in thermal hysteresis, especially the transition between the fluid and hexagonal phases in PE, which is reportedly impeded by oleic acid, LA, and ALA [515,516]. Stearic acid and hydroxylated fatty acids induce a modest shift toward a higher melting temperature (the gel-to-fluid phase transition temperature) in bilayers containing C14:0/C14:0-PC [517]. On the other hand, PCs containing DHA exhibit higher melting points than those containing ALA and AA [518]. Despite DHA’s loose packing property [507], the presence of a saturated fatty acid at the *sn*-1 position in a PC molecule may affect its packing stability by altering both intra- and intermolecular van der Waals interactions.

### 4.2. Relative Functional Significance of Polyunsaturated Fatty Acids

The multifaceted role of fatty acids within various membrane lipids, particularly n3-fatty acids, has been documented over the past century. Diets rich in n3-fatty acids have been extensively associated with elevating the proportions of n3-fatty acids in biomembranes, thereby contributing to the maintenance of cardiovascular, vascular, and neural health [519,520]. Moreover, these dietary choices have shown promise in ameliorating conditions such as atherosclerosis, hypercholesterolemia, and cancer [487,521,522]. Fatty acids exhibit a multitude of physicochemical properties that serve diverse purposes by modifying the characteristics of bilayer lipids, thus influencing signal transduction. Notably, the length of a fatty acid exerts a marked influence on cellular signaling and metabolic processes. For instance, SLs containing short-chain fatty acids have been observed to augment susceptibility to apoptosis [523]. Membrane lipids enriched with monounsaturated fatty acids also play specific functional roles. Cao et al. [524] have reported that palmitoleate can function as a lipid-regulating hormone, often referred to as a ‘lipokine’, by enhancing sensitivity to glucose and inhibiting lipogenesis and hepatic inflammation. Furthermore, the well-documented antitumor and apoptotic properties of C18-monounsaturated fatty acids in carcinoma cells [525] underscore their potential utility in anticancer medications.

The acyl chain length of SLs, particularly Cers, significantly influences TAG accumulation and the hepatic uptake of fatty acids, which is attributed to the disruption of CD36/FAT expression [526]. This discovery underscores the role of CerS2 in catalyzing the generation of very long-chained Cers. In a cardiac context, long-saturated and polyunsaturated fatty acids have been shown to up-regulate voltage-dependent calcium release in cardiac myocytes [527], implicating their involvement in cardiac damage. Sassa and Kihara (2014) have presented a comprehensive review detailing the metabolism of very long-chain fatty acids and their contributions to the health and pathophysiology of various tissues, including the skin, meibum, retina, testis, and brain. The extensive body of available literature underscores the remarkable significance of polyunsaturated fatty acids, which have been the subject of substantial research due to their diverse bio-functional roles across various cell types.

The essentiality of LA and ALA in mammals transcends their role as diet-derived fatty acids; they are also fundamental precursors for the synthesis of long and very long polyunsaturated fatty acids. However, it is imperative to note that not all absorbed dietary LA and ALA are available for elongation and desaturation processes, as a fraction of these fatty acids are utilized for generating the energy source ATP during the β-oxidation process. Therefore, a prolonged deficiency in LA and/or ALA can lead to severe consequences, often manifesting as clinical symptoms [528,529,530,531,532]. LA, specifically, serves as a critical substrate for the biosynthesis of arachidonic acid (AA) and adrenic acid, both of which play crucial roles in early brain development [533,534,535]. Furthermore, LA is indispensable for the formation of n-hydroxyceramides, which covalently bond with epidermal proteins, thereby curtailing water loss and bolstering the skin’s barrier function [536]. In addition, it has been demonstrated that LA exhibits antibiotic-like properties, manifesting as an antibacterial effect that inhibits microbial adhesion to cells, a characteristic shared by numerous polyunsaturated fatty acids [537,538,539,540].

In general, polyunsaturated fatty acids exert substantial influence over the epidermis and its barrier properties. Notably, dietary supplementation of γ-linolenic acid has demonstrated anti-inflammatory properties [541] and has proven effective in enhancing skin characteristics in a dry skin model by reinforcing the skin’s barrier function and limiting dehydration [542]. Similar observations have been made with the supplementation of EPA and DHA [543], where an increase in the production of specific Cer families with anti-inflammatory properties was evident. It is worth noting that the effects of these fatty acids varied across distinct skin regions, including the epidermis, dermis, and hypodermis [543]. It is of particular interest that, among n6-fatty acids, γ-linolenic acid and DGLA have gained recognition for their anti-inflammatory attributes, similar to those of EPA and DHA (n3-fatty acids). Notably, γ-linolenic acid is found in inflammatory cells at relatively modest concentrations, and increasing its dietary intake does not necessarily lead to a proportional increase in its intracellular levels [544,545]. Given the efficient conversion of γ-linolenic acid to DGLA in mammals, it is conceivable that DGLA-derived lipid mediators play a role in mediating the anti-inflammatory effects associated with γ-linolenic [546].

The essentiality of ALA initially became apparent through observations of its ability to alleviate symptoms related to LA deficiency [547]. Its significance grew further when it was established that ALA serves as a precursor for EPA and DHA [548,549]. These C20 and C22 n3-polyunsaturated fatty acids are known to constitute a significant portion of the membrane lipids in critical tissues such as the brain [550], retina [551], and testis [552], reflecting their involvement in neurotransmission, visual excitation, and spermium maturation. The implications of n3-polyunsaturated fatty acids, especially DHA, on these tissues have been extensively documented in numerous studies [467,553,554,555,556,557,558,559,560,561,562,563,564,565,566]. These studies have proposed numerous biological functions for n3-polyunsaturated fatty acids, including the modulation of membrane proteins, gene expression, neurogenesis, enhancement of microcirculation, learning processes, and cellular protection. Notably, in neural tissue, the selectivity of PS declines under DHA deficiency [420]. The role of n3-fatty acids incorporated into PS in improving memory [567] and protecting against age-related lipid metabolic disorders, especially in the presence of DHA-enriched PC [567], is well acknowledged. For instance, DHA inhibits the production of amyloid-beta (Aβ) peptides associated with cognitive impairments, thereby mitigating amyloidogenesis, oxidative stress, and apoptosis [520]. The overall impact of polyunsaturated fatty acids on oxidative stress remains a subject of debate, as Shefer-Weinberg et al. [568] found that exposure to polyunsaturated fatty acids elevated oxidative stress biomarkers levels. In this context, it is plausible to hypothesize that the diverse polyunsaturated fatty acids may elicit distinct effects. Nonetheless, DHA has been reported to enhance the fluidity of the synaptic plasma membrane and induce the expression of other memory-related proteins [465]. Consequently, n3-fatty acids, particularly DHA, have gained significant scientific interest, leading to the development of nutraceuticals in the form of dietary supplements that incorporate these fatty acids.

In the preceding sections, the various roles of DHA in the physicochemical properties of membranes have been described. However, DHA also has crucial biological functions within membranes. DHA-enriched membranes have been suggested to influence membrane proteins by inducing curvature stress [569,570,571], affecting membrane thickness [473,570,572], and modulating fatty acid packing free volume [565]. These alterations in membrane properties can lead to modifications in the activity of most cellular proteins, affecting signal propagation. For instance, unsaturated fatty acids have been reported to interact with various proteins, including rhodopsin, ion channels (L-type Ca^2+^ and Na^+^), protein kinase C (PKC), apoptosis-associated proteins, PPAR-γ, nuclear receptor Nur77, G-protein coupled receptor 40, mitogen-activated protein kinase, toll-like receptors, and nuclear factor kappa-light-chain-enhancer of activated B cells (NF-κB) [573,574,575,576,577,578,579,580,581,582,583,584,585]. However, the major relationships between DHA and cellular protein activities remain ambiguous due to the vast diversity of proteins, the complexity of protein interactions, and the limited number of studies. Despite being highly unsaturated (with six double bonds), DHA exhibits antioxidant properties in the liver [586], brain [587,588], and skeletal muscles [465]. This property is of particular significance for fertility, as Roqueta-Rivera et al. [589] observed that DHA supplementation effectively restored impaired spermatogenesis in male mice.

Both EPA and DHA have demonstrated the ability to counteract pro-inflammatory cytokines by down-regulating the NF-κB signaling pathway [590,591,592], a transcriptional pathway that regulates both innate and adaptive immune responses. In contrast, AA levels have been found to correlate positively with lipid peroxidation [593] and activation of the NF-κB signaling pathway [594], thereby promoting pro-inflammatory stimuli. AA can also up-regulate SMase activity [595], leading to increased levels of Cers, molecules that trigger apoptotic signals, which are derived from SM hydrolysis. Thus, AA is a biologically essential fatty acid, contributing to a wide array of functions either directly or through its bioactive metabolites. Hashidate-Yoshida et al. [596] demonstrated that AA facilitates the transportation of triglycerides to the lumen of the ER in hepatocytes and enterocytes.

The ratio between fatty acids within cellular membranes serves as a reflection of universal cellular signaling and inflammatory responses. Notably, EPA and DHA exhibit distinct signaling profiles compared to AA. Consequently, the ratio of EPA and DHA to AA can serve as an indirect indicator for assessing the inflammatory response and lipid peroxidation. It is worth emphasizing that these fatty acids serve as precursors for numerous bioactive mediators, contributing to a wide array of physiological functions. However, it is also important to recognize that many of the reported findings are likely attributed to the direct alterations of membrane physicochemical properties and membrane-associated proteins [481], along with the unidentified bioactive metabolites they generate. Polyunsaturated fatty acids can undergo chemical reactions with various molecules and cellular components, resulting in the formation of novel compounds with biological activity. Heshmati [597] has described interactions between n3-fatty acids and specific transcription factors in genes. Furthermore, an intriguing observation is the interaction of nitric oxide (NO) with polyunsaturated fatty acids, leading to the formation of nitroalkene derivatives. These plasma-identifiable derivatives have been demonstrated to promote vascular relaxation, inhibit neutrophil cell degranulation and superoxide production, and hinder platelet activation [598,599,600]. Nitroalkene derivatives possess inherent PPAR ligand activity and are known to degrade into NO in the bloodstream. These observations underscore the capacity of polyunsaturated fatty acids to engage in reactions with other non-lipidous cellular constituents, resulting in the formation of novel compounds with specific biological activities.

### 4.3. Bioactive Lipid Mediators Derived from Fatty Acids

Numerous classes of lipids, including LysoP, SLs, PA, DAG, inositol phosphate, *N*-acylethanolamine, fatty acids, and oxylipins, are renowned for their bioactive intracellular and extracellular signaling properties, acting as messengers/mediators. On the other hand, certain functions of polyunsaturated fatty acids necessitate their conversion into lipid mediators. These mediators serve as signaling molecules that modulate various biological processes, including the inflammatory response, gene transcription, and signal transduction pathways. For instance, the tissue hormone-like lipids referred to as “eicosanoids”, which were initially identified in the prostate [601], possess the ability to regulate the function of various transcription factors, thus inducing alterations in gene expression. To comprehend the mechanisms underlying the generation of lipid mediators derived from fatty acids, this section elaborates on the cleavage mechanism of membrane fatty acids, the oxygenation mechanisms of deacylated fatty acids, and the biological functions of lipid mediators derived from fatty acids.

#### 4.3.1. Enzyme-Mediated Cleavage of Fatty Acids from Membranes

Polyunsaturated fatty acids are abundant in biomembranes but can be enzymatically cleaved from *sn*-positions and *N*-acyl linkages of membrane lipids by lipase-type enzymes. These enzymes encompass PLA2, phospholipase B (PLB, an enzyme with both PLA1 and PLA2 activities), diacylglycerol lipase [602,603], CDase [604], glucosylceramide deacylase [605,606], and sphingomyelin deacylase [605,607]. Other phospholipase enzymes, such as PLA1, PLC, and PLD, play a lesser role in the generation of polyunsaturated fatty acid-derived mediators, as they cleave the highly saturated chains at the *sn*-1 position [608], the phosphate group at the *sn*-3 position [609], and the head group from the phosphorus group [610], respectively.

Among these enzymes, PLA2 has received considerable attention due to the biological importance of its substrates. Over recent decades, six isoforms of PLA2, which hydrolyze the ester bond at the *sn*-2 position, have been identified [611]. Each of these isoforms exhibits selectivity for specific fatty acids on phospholipids. For example, cytosolic PLA2α (cPLA2α) acts on phospholipids rich in AA [612], calcium-independent PLA2β (iPLA2β) acts on phospholipids rich in DHA [613,614], and secretory PLA2 (sPLA2) acts on phospholipids containing various fatty acids, including AA, EPA, and DHA [615,616]. On the other hand, PLB possesses both hydrolase activity, cleaving ester bonds on the *sn*-1 and *sn*-2 positions of phospholipids, and acyltransferase activity, acylating fatty acid to form LysoP, and, as a result, may contribute less to the production of oxylipins compared to PLA2. Following the removal of fatty acids from complex membrane lipids, various events, including reacylation and/or oxidation, may occur.

#### 4.3.2. Fatty Acid Oxygenation

CHOL and liberated polyunsaturated fatty acids can undergo oxidation through enzymatic processes and non-enzymatic agents, such as reactive oxygen species (ROS). This oxidative transformation leads to the production of oxysterols and oxylipins, respectively. Notably, these compounds may also originate from dietary sources. It is of particular significance that polyunsaturated fatty acids frequently engage in metabolic competition with each other, a phenomenon specifically mediated by enzymes such as PLA2 and oxidative enzymes. The extent of competition among different fatty acids depends on their respective concentrations within the cell and their relative affinities for oxidative enzymes and reactive molecules [617,618]. These oxidized lipid metabolites serve as pivotal mediators in cell signaling. For instance, oxysterols have the capacity to interact with nuclear receptors and, as a consequence, modulate gene expression [619,620]. This section primarily focuses on the enzymatic pathways involved in generating these bioactive lipid mediators.

**Figure 7:**
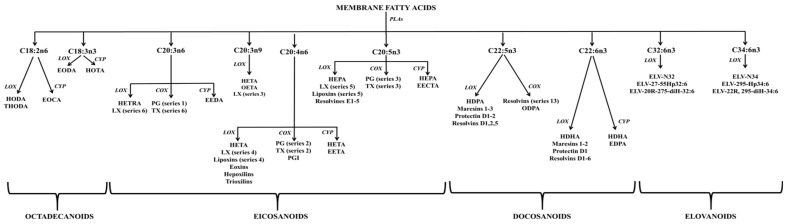
Diagram illustrating the various lipid mediators (including octadecanoids, eicosanoids, docosanoids, and elovanoids) synthesized from fatty acids such as LA, ALA, DGLA, Mead acid, AA, EPA, DPA-n3, DHA, C32:6n3, and C34:6n3. Abbreviations: COX, cyclooxygenase; EDPA, epoxy-docosapentaenoic acid; EECTA, epoxy-ecosatetraenoic acid; EEDA, epoxy-eicosadienoic acid; EETA, epoxy-eicosatrienoic acid; ELV, elovanoids; EOCA, epoxy-octadecenoic acid; EODA, epoxy-octadecadienoic acid; CYP, cytochrome P450; HDHA, hydroxy-docosahexaenoic acid; HDPA, hydroxy-docosapentaenoic acid; HEPA, hydroxy-eicosapentaenoic acid; HETA, eicosatetraenoic acid; HETRA, hydroxy-eicosatrienoic acid; HODA, hydroxy-octadecadienoic acid; HOTA, hydroxy-octadecatrienoic acid; LOX, lipoxygenase; LX, leukotrienes; ODPA, oxodocosapentaenoic acid; OETA, oxoeicosatetraenoic acid; PG, prostaglandin; PGI, prostacyclins; PLAs, phospholipases; THODA, trihydroxy-octadecenoic acid; TX, thromboxane.

Numerous bioactive oxylipins have been identified as products of enzymatic pathways, including those facilitated by cyclooxygenase (COX) and subsequent synthases, lipoxygenase (LOX), and cytochrome P450 (CYP) mixed-function oxidase enzymes [621]. These oxylipins are further categorized based on the chain length of their respective substrates (see Figure 7), resulting in octadecanoids (derived from C18 fatty acids), eicosanoids (derived from C20 fatty acids), docosanoids (derived from C22 fatty acids), and elovanoids (derived from C32 or C34 fatty acids).

In mammals, COXs, also known as housekeeping enzymes, comprise three isoforms as follows: COX-1, COX-2, and COX-3 isoforms [622], with COX-3 being considered a variant of COX-1 [623]. These enzymes are heme-containing and possess the dual capacity to function as both oxygenases and peroxidases. Notably, these enzymes are constitutively expressed and are subject to modulation by inflammatory signals. Their main role involves catalyzing the oxygenation of various unsaturated fatty acids, culminating in the generation of bioactive end-products collectively referred to as prostanoids. These prostanoids encompass the prostaglandin series (PGD, PGE (dinoprostone), PGF (carboprost), and PGI (prostacy-clins)), thromboxanes, hydroxy fatty acids, resolvins (series 13), and oxo-fatty acids [624,625].

LOXs, which comprise six genes within the human genome, represent a class of non-heme iron-containing dioxygenases. These enzymes possess the capability to oxygenate a broad range of unsaturated fatty acids. It is noteworthy that LOX enzymes typically exist in an inactive form at their base state, necessitating activation facilitated by hydroperoxides. Subsequently, they act on a diverse array of substrates and engage in various modes of action, including dioxygenase activity, functioning as catalysts in processes characterized by the involvement of free radicals [626,627]. These catalytic actions lead to the formation of bioactive end-products recognized as hydroperoxyl fatty acids and their metabolites, including leukotrienes, lipoxins, resolvins, protectins, maresins, and elovanoids [628,629].

On the other hand, CYPs are enzymes encoded by an extensive set of up to 57 genes within the human genome, representing a class of monooxygenases widely distributed in mammals. These enzymes exhibit elevated activity levels in numerous tissues, including but not limited to the liver, brain, kidneys, and lungs [629,630]. CYPs are renowned for their involvement in various modes of action, including hydroxylation, heteroatom oxidation, allylic oxidation reactions, group migration, and various other enzymatic reactions [631,632,633]. They display the capability to act on a diverse range of unsaturated fatty acids and sterols [629], thereby generating a wide array of lipid mediators. In particular, these lipid mediators consist of hydroxyl and epoxy fatty acids, which play critical roles in the induction of various signaling pathways.

#### 4.3.3. Functions of Bioactive Lipid Mediators

Bioactive lipid mediators go beyond being inert components of cellular membranes. Instead, they serve as dynamic signaling agents and are capable of modulating a wide range of signaling pathways, gene regulation, and immune responses. The unique characteristics and functions of these mediators have led to extensive research efforts aimed at harnessing their therapeutic potential for developing innovative treatment/preventive approaches. Therefore, comprehending the functions of bioactive lipid mediators holds great importance in the domains of biological and medical research.

##### Octadecanoids

**Figure 8:**
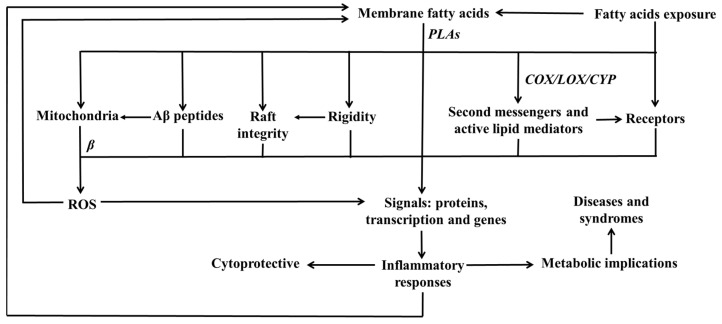
Illustration of the influence of bilayer-cleaved fatty acids on cellular signaling pathways and inflammation responses. Abbreviations: *β*, β-oxidation; COX, cyclooxygenase; CYP, cytochrome P450; LOX, lipoxygenase; PLAs, phospholipases; ROS, reactive oxygen species.

Essential fatty acids and their extended metabolites have well-documented diverse biological effects and implications in various disease mechanisms. The effects of lipid mediators can vary depending on the type of cells and metabolic factors, leading to both beneficial and detrimental metabolic outcomes (see Figure 8).

LA and ALA play an essential role in the generation of lipid mediators. As essential fatty acids, dietary levels of LA and ALA contribute to their proportions within cellular membranes, potentially playing a crucial role in modulating the extent/degree/severity of inflammation development. Epoxy-octadecadienoic acid and hydroxy-octadecatrienoic acid are lipid mediators derived from ALA through the enzymatic actions of LOX and CYP, respectively [624]. However, further research is needed to fully comprehend the bioactive functions of octadecanoids derived from ALA. Notably, Kumar et al. [634] have suggested that these mediators primarily exert anti-inflammatory effects. On the other hand, oxidized LA metabolites, including hydroxy, trihydroxy, and epoxy fatty acids, are produced under the effects of LOXs and CYPs [546,624]. These metabolites have been implicated in various biological pathways, including brain dysfunction [635], the inhibition of platelet adhesion in endothelial cells [636], the induction of inflammation signals [637,638,639], the maintenance of skin barrier integrity [536], the inhibition of pain thresholds [640,641], and the promotion of metabolic syndromes and cancer [638,642]. Consequently, these LA-derived mediators may greatly contribute to the inflammatory processes and the progression of diseases.

Both LA and ALA serve as essential fatty acids and precursors for extended polyunsaturated fatty acids. Thus, their dietary concentrations can alter the levels of long and very long chain polyunsaturated fatty acids within cellular biomembranes. However, the extent of this influence may vary depending on the specific substrate and metabolic pathways. Notably, a high dietary intake of LA tends to not significantly elevate the proportion of AA or the associated inflammatory cascades in humans [643]. In contrast, a high dietary intake of ALA has been shown to increase EPA and DHA concentrations [644]. Nevertheless, it is essential to recognize that the de novo pathways for elongating essential fatty acids exhibit variations among species. For instance, the conversion rate of ALA to its extended polyunsaturated fatty acids is lower in humans [645] than in marine species. These findings underscore the potential variability in the biological functions of ALA, with specific implications in distinct species to fulfill particular physiological functions.

##### Eicosanoids

Eicosanoids are bioactive lipid mediators primarily derived from unesterified fatty acids and are characterized by their autocrine/paracrine hormone activities. They mediate local signals and reactions, including processes related to homeostasis, inflammation, and anti-inflammation. Eicosanoids comprise various structures, such as PGs, thromboxanes, leukotrienes, lipoxins, and resolvins. Despite the fact that most mammalian cells are capable of synthesizing eicosanoids, the specific pathways and responses can vary by cell type [646]. DGLA, rapidly extended from γ-linolenic acid, serves as a substrate for enzymes such as COX, which yields series 1 prostaglandins and thromboxanes, 15-LOX, which yields 5-hydroxyeicosatrienoic acid, and CYP, which yields epoxy-eicosadienoic acid. Eicosanoids derived from DGLA are generally considered to be anti-inflammatory [647,648].

On the other hand, beyond the role of AA as a polyene fatty acid, it is unquestionably crucial in biomembranes as it is the primary target for most membrane-modifying effects. The activation of the PLA2 enzyme, which is responsible for cleaving AA from membrane phospholipids, often leads to membrane injury. According to Samuelsson [649], this enzyme rapidly (within seconds to minutes) responds to acute stimuli, releasing AA from membrane lipids. Liberated AA can be utilized as a precursor for the production of eicosanoids under the effects of COX (generating series 2 prostaglandins, prostacyclins, and thromboxanes), LOX (generating leukotrienes, lipoxins, eoxins, hepoxilins, and trioxilins), and CYP (generating hydroxyeicosatetraenoic acid and epoxyeicosatrienoic acid) [546,624]. AA-derived eicosanoids, often referred to as arachidonate or eicosanoid cascades, are involved in multiple systems, including vascular, inflammatory, renal, and neuronal signaling, as well as angiogenesis [650]. For instance, AA-derived eicosanoids have been shown to increase the permeability of the blood–brain barrier in humans [651], revealing the potential for drug modulation of this barrier.

Eicosanoids derived from Mead acid have displayed anti-inflammatory properties. For instance, oxygenated products of Mead acid via 5-lipoxygenase are produced during inflammation, providing potent activities [652]. However, the exact roles of this fatty acid are not yet clearly defined [652,653,654,655], necessitating further research. On the other hand, LOXs oxidize EPA to produce resolvins [656], which are renowned for their anti-inflammatory properties. EPA can also undergo oxygenation via COXs (yielding hydroxy-eicosapentaenoic acid and epoxy-eicosatetraenoic acid) and CYPs (yielding series 3 prostaglandins and leukotrienes) [624,657]. Overall, EPA-derived eicosanoids exhibit anti-inflammatory stimuli, such as the inhibition of platelet aggregation [658].

Eicosanoids play a remarkable role in the regulation of inflammatory responses by modulating pro-inflammatory cytokines, chemokines, and other signaling molecules. They have the potential to influence the recruitment, activation, and function of immune cells. However, it is essential to recognize that eicosanoids can exhibit both pro-inflammatory and anti-inflammatory effects, with the ultimate effect determined by various factors, including mediator concentrations, timing of production, and the sensitivity of targeted cells/tissues [460,659]. Commonly, eicosanoids derived from different fatty acids, such as AA, Mead acid, and EPA, exhibit strikingly distinct biological effects, despite their closely resembling molecular structures.

Eicosanoids derived from n3-fatty acids are well-recognized for their anti-inflammatory properties, while those originating from n6-fatty acids are generally considered to be pro-inflammatory [660,661]. However, it is important to note that not all n6-fatty acids exert pro-inflammatory effects. Some prostanoids (PGs and thromboxanes), n6-fatty acid-derived lipoxins, as well as mediators derived from γ-linolenic and DGLA, along with adrenic acid, have been found to express anti-inflammatory properties and cytoprotective actions [662,663,664,665,666,667,668,669]. Imbalances in the production of eicosanoids have been implicated in numerous pathological processes, including inflammation, autoimmunity, allergy, cancer, atherosclerosis, and metabolic and degenerative diseases [650], by disrupting the normal lipid signaling pathways. In light of this, strategies that involve the suppression of COX, LOX, and CYP enzymes, which are responsible for the synthesis of active lipid mediators, may hold therapeutic potential for the management of disease-related inflammation and oxidative stress.

##### Docosanoids

DHA, likely the reason for the biological necessity of ALA, is a very long polyunsaturated fatty acid that accumulates abundantly in crucial tissues such as the brain, retina, and testis. Though EPA is known to produce pre-resolving mediators (resolvins), it is DHA that serves as the major precursor for these compounds [656,670]. Specialized pro-resolving mediators (SPMs), known as docosanoids, are primarily derived from the LOX oxidation of DHA and DPA-n3 [670,671,672,673]. However, COX activity on DPA-n3 can also generate SPMs [672], and CYP activity on DHA yields hydroxy-docosapentaenoic acid and epoxy-docosapentaenoic acid [624]. The pre-resolving family comprises various structures, including resolvins, docosatrienes, maresins, and protectins, all of which exhibit anti-inflammatory and pro-resolving properties, countering the effects of pro-inflammatory cascades [659,666,667,671,672,673,674,675,676,677,678,679,680,681,682]. These docosanoids, which are derived from DPA-n3 and DHA, play pivotal roles in the regulation of leukocyte trafficking, suppression of cytokine expression, inhibition of brain ischemia-reperfusion injury, maintenance of cellular homeostasis, mitigation of potential DNA oxidation, normalization of brain-derived neurotrophic factor levels, and promotion of the clearance of apoptotic cells and cellular debris by phagocytes. Thus, these mediators represent a promising therapeutic approach for resolving cellular inflammation and associated diseases.

Furthermore, EPA and DHA are known to limit pro-inflammatory cytokines and reduce inflammation, potentially by increasing peroxisome proliferator-activated receptor alpha (PPAR-α) mRNA and protein activities [683]. Remarkably, alternative lipid mediators with resembling impacts to resolvins have been identified. According to Dalli et al. [684], DPA-n3, an intermediate fatty acid during DHA synthesis, is transformed into novel immunoresolvents similar to resolvins in mice and human leukocytes during inflammation. However, it is important to acknowledge that the resolution of inflammation mediated by docosanoids is characterized by its complexity in restoring cellular homeostasis [656].

##### Elovanoids

In response to unmitigated oxidative stress, elovanoids exhibit a remarkable ability to enhance the intracellular synthesis of pro-survival signals, owing to their distinctive molecular structures. This class of bioactive lipids, initially discovered by Bazan’s research group in the retinal pigment epithelium in 2017 [685], is derived from mono-hydroxyl-very long polyunsaturated fatty acids formed through the enzymatic activity of ELVOL4 and LOX. It is important to emphasize that very long polyunsaturated fatty acids are prominent constituents of critical tissues such as the brain, testis, and spermatozoa [686]. This observation suggests the potential formation of elovanoids in these tissues, where they might serve as mediators of specific signals. However, while the retina has been a focal point of research on elovanoids, studies examining neural signaling are comparatively limited.

Elovanoids play an indispensable role in the functions of the retina and neural signaling [290,685,687,688,689,690,691]. The protective effects of elovanoids in these tissues are most likely attributed to their role in mitigating the effects of oxidative stress. In events where oxidative stress remains unresolved, elovanoids serve as critical survival signals [685]. These authors have reported that dihydroxylated derivatives of C32:6n-3 and C34:6n-3 effectively protect retinal pigment epithelial cells from apoptosis induced by hydrogen peroxide. These derivatives have been shown to up-regulate the expression of pro-survival proteins, including Bcl-2 and Bcl-xL, while concurrently down-regulating the expression of pro-apoptotic proteins, such as Bax, Bim, and Bid. These findings underscore the ability of elovanoids to mitigate the cytotoxic effects of ROS on photoreceptor cells and contribute to their survival.

## 5. Conclusions and Future Perspectives

This review intends to provide an in-depth overview of the lipids of eukaryotic cell membrane lipids, with a particular emphasis on fatty acids. It introduces the extensive array of lipids present in biomembranes and delves into their composition within healthy organisms, thereby illustrating the intricate nature of lipid metabolism and its fundamental role within cells. This perspective underscores the remarkable adaptability and flexibility inherent in the fatty acid profiles of biomembranes, enabling organisms to rapidly respond to various stimuli, including alterations in environmental temperature, dietary factors, inflammatory processes, or diseases. Thus, the absence of a universally defined “physiologically normal fatty acid composition” underscores the natural variability in fatty acid composition. This natural phenomenon is, likewise, a continuous process of adaptation. This review further provides an in-depth exploration of fatty acid biosynthesis and post-synthetic modifications, such as elongation and desaturation. In addition, it highlights the preferences of fatty acids for incorporation into diverse complex membrane lipids and their roles in biological systems, encompassing both physicochemical properties and the regulation of biological signaling. This understanding holds significant implications across various disciplines, including lipid-based drug delivery, cell membrane engineering, and the advancement of lipid-based biomaterials. Nevertheless, further research remains essential to unveil the intricate mechanisms and regulatory pathways governing eukaryotic lipid metabolism and fatty acid composition. This includes investigations into the mechanisms underpinning cellular membrane adaptability, with the potential to shed light on the molecular foundations of cellular processes, diverse diseases, and the development of therapeutic strategies for lipid-related disorders.

Evidently, the pivotal role of fatty acids in biomembranes is ascending and is poised to exert a substantial influence across various disciplines, notably within the realms of nutrition and medicine. This review serves to illuminate the multifaceted roles and contributions of distinct membrane lipids, along with their associated fatty acids, with a specific focus on matters pertaining to health and the intricate aspects of inflammatory responses. Enhancing our comprehensive comprehension of the diverse repertoire of membrane lipids stands to be invaluable for assessing the overall health of organisms. The trajectory of the field nutrition is set to emphasize progressively specific fatty acids that are indispensable for organism health. In this context, the n3 and n6-fatty acids are assuming paramount significance due to their critical roles as precursors for bioactive lipids that play a pivotal role in the modulation of inflammatory processes. They also contribute indispensably to the development and sustenance of vital organ functions, exemplified by the brain, heart, lungs, liver, and kidneys. Elevated levels of these fatty acids have been consistently correlated with to the regulation of chronic maladies, encompassing diabetes, cardiovascular disorders, and certain forms of cancer. However, it is crucial to recognize that the optimization of fatty acid biosynthesis, the preservation of their stability, and a comprehensive understanding of their various roles in biological systems continue to remain areas ripe for exploration. Thus, the unwavering dedication to research and development in this domain holds the promise of unveiling the unlocking of novel approaches to incorporate these essential nutrients into the diets of organisms, thereby fostering enduring health and well-being.
